# Designer diatom episomes delivered by bacterial conjugation

**DOI:** 10.1038/ncomms7925

**Published:** 2015-04-21

**Authors:** Bogumil J. Karas, Rachel E. Diner, Stephane C. Lefebvre, Jeff McQuaid, Alex P.R. Phillips, Chari M. Noddings, John K. Brunson, Ruben E. Valas, Thomas J. Deerinck, Jelena Jablanovic, Jeroen T.F. Gillard, Karen Beeri, Mark H. Ellisman, John I. Glass, Clyde A. Hutchison III, Hamilton O. Smith, J. Craig Venter, Andrew E. Allen, Christopher L. Dupont, Philip D. Weyman

**Affiliations:** 1 Synthetic Biology and Bioenergy Group, J. Craig Venter Institute, La Jolla, California 92037, USA; 2 Microbial and Environmental Genomics Group, J. Craig Venter Institute, La Jolla, California 92037, USA; 3 Integrative Oceanography Division, Scripps Institution of Oceanography, University of California San Diego, La Jolla, California 92037, USA; 4 National Center for Microscopy and Imaging Research, University of California, San Diego, La Jolla, California 92093, USA

## Abstract

Eukaryotic microalgae hold great promise for the bioproduction of fuels and higher value chemicals. However, compared with model genetic organisms such as *Escherichia coli* and *Saccharomyces cerevisiae*, characterization of the complex biology and biochemistry of algae and strain improvement has been hampered by the inefficient genetic tools. To date, many algal species are transformable only via particle bombardment, and the introduced DNA is integrated randomly into the nuclear genome. Here we describe the first nuclear episomal vector for diatoms and a plasmid delivery method via conjugation from *Escherichia coli* to the diatoms *Phaeodactylum tricornutum* and *Thalassiosira pseudonana*. We identify a yeast-derived sequence that enables stable episome replication in these diatoms even in the absence of antibiotic selection and show that episomes are maintained as closed circles at copy number equivalent to native chromosomes. This highly efficient genetic system facilitates high-throughput functional characterization of algal genes and accelerates molecular phytoplankton research.

## Abstract (web summary)

Algae hold great promise for biofuel and chemical production but their use as model systems is hampered by the absence of suitable genetic tools. Here Karas *et al*. present a nuclear episomal vector for diatoms that is maintained in the absence of antibiotics, and a plasmid delivery method via conjugation with *E. coli*.

## Graphical abstract

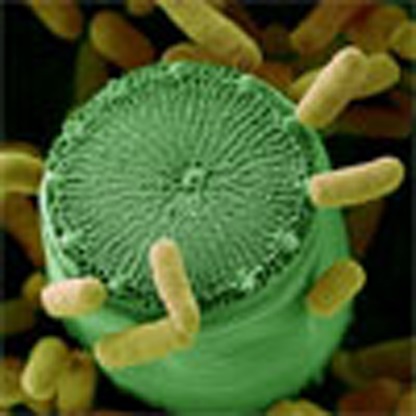

Diatoms are eukaryotic phytoplankton that contribute a significant fraction of global primary productivity and demonstrate great potential for autotrophic bioproduction of fuels and higher value chemicals^1^,^2^,^3^. Although methods for genetic manipulation currently exist for some diatom species^4^,^5^,^6^,^7^,^8^, they are slow compared with the efficient methods available for other model microbes such as *E. coli* and yeast, and this has stymied both basic diatom research and applied strain development. To accelerate research in ecologically and biotechnologically important microalgae, we sought to develop diatom episomal vectors and improved transformation methods.

Circular DNA molecules have been previously isolated from diatoms, but they have never been successfully reintroduced as episomes^9^,^10^. This is unfortunate because episomes provide a reliable, consistent and predictable platform for protein expression by avoiding the complications of random chromosomal integration including multiple insertions, position-specific effects on expression and potential knockout of non-targeted genes^4^,^7^,^8^. Consistent protein expression from episomes may allow for efficient complementation experiments of diatom mutants made with recently developed TALEN technology^11^,^12^.

Episomes can be efficiently moved among bacteria and even between bacteria and eukaryotes via conjugation. Reports of transkingdom conjugation in other systems^13^,^14^,^15^,^16^ motivated us to explore direct conjugation of DNA into the pennate diatom *Phaeodactylum tricornutum* as an alternative to biolistic transformation methods that are standard for many diatom species^4^,^5^,^6^. A conjugative link from *Escherichia coli* to diatoms streamlines the genetic manipulation workflow for diatoms. Plasmids can be assembled and manipulated *in vitro* before being introduced in *E. coli* for direct conjugative transfer to diatoms, thus eliminating the need for time-consuming, high-yield plasmid DNA preparations and access to expensive specific equipment and reagents (for example, gene gun). In conjugative systems, a two-plasmid system is often used where a conjugative plasmid (for example, RP4/RK2 or its derivatives such as pTA-MOB^17^) contains all of the genes required for establishing a conjugative bridge between the donor and recipient cell and a cargo plasmid contains the construct of interest. Inclusion of an origin of transfer (*oriT*) on the cargo plasmid allows it to be mobilized to the recipient by proteins encoded on the conjugative plasmid. Maintenance of the cargo plasmid as an episome in the recipient cell further requires the presence of sequences that permit replication and segregation of the DNA molecule.

In this paper we describe our approach to identify a DNA sequence that can support episomal replication in the diatoms *Phaeodactylum tricornutum* and *Thalassiosira pseudonana*. Although we had initially intended to isolate a *P. tricornutum* sequence that supported episome replication in diatoms, we identified a sequence encoded by a yeast-derived sequence on the cloning vector that performed this function. We further developed a conjugation-based method to directly transfer episomes from *E. coli* to diatoms. These novel tools and methods compose an efficient and high-throughput system for diatom genetic manipulation that will enable rapid and fundamental advances in diatom functional genetics.

## Results

### Design of episomal vectors

**Figure 1:**
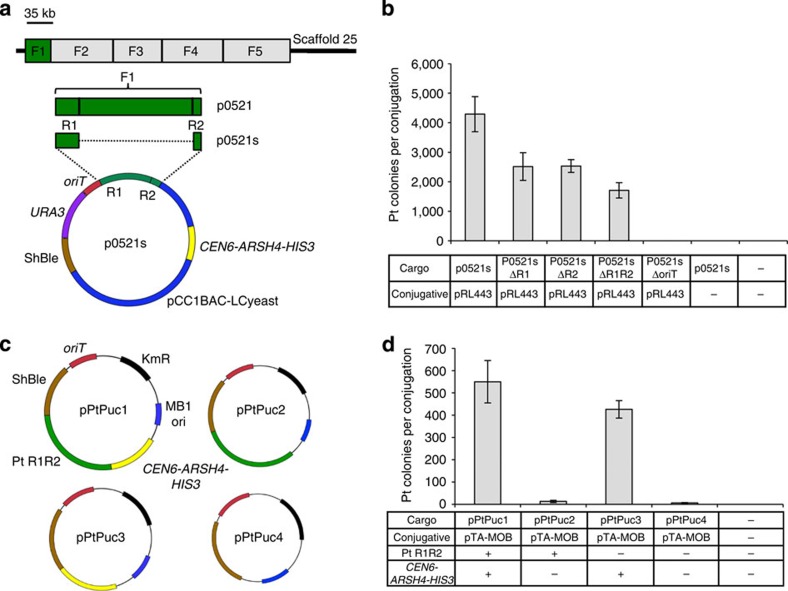
Conjugative transfer of plasmids from *E. coli* to *P. tricornutum*. (**a**) Map of the plasmids p0521 and p0521s and their derivation from *P. tricornutum* scaffold 25. *OriT*, origin of transfer; *URA3*, gene encoding orotidine 5′-phosphate decarboxylase from *S. cerevisiae*, and ShBle, phleomycin-resistance cassette with *P. tricornutum FcpF* promoter and *FcpA* terminator. (**b**) Average number of *P. tricornutum* colonies obtained per conjugation for different ‘cargo' plasmid variants of p0521s. Deletions of features indicated by Δ, pRL443 is the RP4 conjugative plasmid. (**c**) Maps of plasmids used to test the importance of the *P. tricornutum*- and yeast-derived regions. Plasmids pPtPuc1-4 differ in whether they have the *P. tricornutum*-derived region from p0521s (Pt R1R2, green) or the yeast-derived *CEN6-ARSH4-HIS3* (yellow). Other plasmid features present in all four plasmids are described on the pPtPuc1 map. (**d**) Number of *P. tricornutum* colonies obtained after conjugation with the pPtPuc plasmids (‘cargo' plasmids). Features of each plasmid are noted in the table under the figure (for example, the *P. tricornutum*-derived region from p0521S, Pt R1R2, or the yeast elements, *CEN6-ARSH4-HIS3*). Error bars denote one s.d. of the mean from at least three biological replicates per experiment.

To develop an extra-chromosomal replicating vector for diatoms, we first isolated a sequence that functions as a centromere or origin of replication in *P. tricornutum*^18^,^19^. Classical centromere signatures, based on DNA composition^20^,^21^,^22^, are absent from putative full-length *P. tricornutum* chromosomes. Therefore, we implemented an experimental workflow based on iterative transformation of *P. tricornutum* with large, cloned fragments (24–94 kb) of scaffold 25 (Fig. 1a), which is fully assembled between telomeres. Molecules maintained as episomes in *P. tricornutum* could be extracted and successfully reintroduced into *E. coli* by electroporation (a technique we define as ‘episome rescue' henceforth). Episome rescue cycles were performed by (1) growing diatom exconjugant colonies in small liquid cultures, (2) extracting diatom DNA, (3) electroporating *E. coli* with the diatom DNA and (4) extracting and analysing plasmids from *E. coli* using standard techniques (Supplementary Fig. 1).

Several diatom colonies were obtained from transformations with the scaffold 25 fragments using electroporation- and polyethylene glycol (PEG)-mediated transformation methods to prevent shearing of the large plasmids. *P. tricornutum* transformation efficiency using the PEG-mediated methods was low and often yielded only one colony per transformation with an approximate efficiency of 1 × 10^−8^. Of the episomes rescued from these colonies (Supplementary Fig. 1c), we chose to work with plasmids p0521o containing the *P. tricornutum* region 25-1, and a spontaneously minimized version, p0521o-reduced; both plasmids were rescued successfully after a second round of *P. tricornutum* transformation (Supplementary Fig. 1d,e). We modified plasmids p0521o and p0521o-reduced by adding a cloning site composed of the *URA3* gene flanked by I-CeuI and I-SceI sites (see Supplementary Table 1 for primer sequences) and renamed them p0521 and p0521s, respectively. The additional *URA3* marker provides an efficient counter selection to insert DNA sequences into the plasmids using yeast assembly methods.

### Development of conjugation to diatoms

We improved the delivery of p0521 and p0521s to *P. tricornutum* by developing a conjugation-based method that transferred p0521s at an efficiency of 4.0 × 10^−4^ diatom cells, significantly higher than our attempts at electroporation and PEG-mediated transformation (Supplementary Table 2), and higher than reported electroporation^23^ and particle bombardment^6^ efficiencies (4.5 × 10^−5^–10^−7^). Transkingdom conjugation has been demonstrated previously for yeasts and mammalian cells^13^,^14^,^15^,^16^, but never before in the Stramenopile lineage. To verify that conjugation was the mechanism of gene transfer, we performed two important controls. First, phleomycin-resistant *P. tricornutum* colonies were only obtained when *E. coli* contained the conjugative plasmid (RP4 variant pRL443 (ref. 24)), and second, the origin of transfer (*oriT*) on the mobilizable cargo plasmid (for example, p0521s) was essential to obtain phleomycin-resistant *P. tricornutum* colonies (Fig. 1b). Physical association between *E. coli* and diatoms, a necessary prerequisite for conjugation, was identified by scanning electron microscope analysis (Supplementary Fig. 2).

We verified that rescued episomes were from *P. tricornutum* and not from possible leftover *E. coli* used during the conjugation process. Streaking *P. tricornutum* exconjugant cultures on LB plates and incubating at 37 **°**C did not yield any *E. coli* colonies. This effectively confirmed that *E. coli* culture containing the plasmid donor was eliminated during selection of *P. tricornutum* exconjugants. After episome rescue, ∼30% of *P. tricornutum* lines yielded plasmids of the same size as the original starting plasmid (Supplementary Fig. 3). We identified one class of incorrectly sized plasmids as chimeras between the conjugative and the cargo plasmids (RP4 and p0521s, respectively). Chimeras resulting from multiple copies of *oriT* have been previously noted^17^, and we later eliminated them by using a variant of the RP4 conjugative plasmid lacking *oriT* (pTA-MOB^17^). A second class of incorrectly sized plasmids was occasionally recovered with minor size differences (Supplementary Fig. 3). Although sequencing of several plasmids identified small deletions and a retrotransposon element insertion event (Supplementary Fig. 4), plasmids were stably maintained once identified as having the correct size as discussed below.

### DNA sequence conferring episomal replication in diatoms

To identify the elements of p0521s allowing replication in diatoms, we engineered several variants either lacking the *P. tricornutum*-derived region or lacking other components of the plasmid. First, we mapped the 2.5-kb *P. tricornutum* sequence in p0521s to two distinct subfragments within fragment 25-1 (Fig. 1a). Compared with conjugation with the full-length p05201s plasmid, deletion of either of the two subfragments (R1 or R2) that make up the 2.5-kb region led to approximately half as many *P. tricornutum* exconjugants per mating (Fig. 1b). Surprisingly, complete removal of the *P. tricornutum*-derived region still yielded exconjugants at high efficiency (Fig. 1b), although plasmid p0521s containing the region yielded ∼2.5-fold more exconjugants than the version with the *P. tricornutum* sequence deleted. Similar numbers of correctly sized plasmids were rescued regardless of the presence of the *P. tricornutum* region (7/20 or 8/20 for p0521s or p0521s-ΔR1R2, respectively)(Supplementary Fig. 3 and Supplementary Table 3). Thus, while *P. tricornutum* fragments increased conjugation efficiency modestly, they were not essential for this process and therefore, another sequence on the cargo plasmid was responsible for replication in *P. tricornutum*.

To identify the non-diatom sequence responsible for replication in *P. tricornutum*, we varied three possible regions of the p0521s plasmid: the bacterial maintenance region and plasmid backbone, the yeast maintenance region (*CEN6-ARSH4-HIS3*), and the *P. tricornutum*-derived region. We tested the influence of the bacterial maintenance region from p0521s (that is, pCC1BAC) by replacing it with the pUC19 plasmid backbone, and used this bacterial replication origin together with the *P. tricornutum* ShBle cassette and *oriT* sequence to make the four test plasmids pPtPuc1 through pPtPuc4 (Fig. 1c). The yeast maintenance region was cloned in plasmids pPtPuc1 and 3, the *P. tricornutum*-derived region was cloned in plasmids pPtPuc1 and 2, and the final plasmid, pPtPuc4, contained only the bacterial maintenance region from pUC19 with the *oriT* and ShBle cassette for phleomycin resistance in *P. tricornutum*.

*P. tricornutum* exconjugants were obtained for all four pPtPuc plasmids; however, pPtPuc1 and pPtPuc3, both containing the yeast maintenance region, *CEN6-ARSH4-HIS3*, yielded over 30-fold more colonies than pPtPuc2 and pPtPuc4 (Fig. 1c and Supplementary Table 3). Episome rescue was successful for 20/20 and 19/20 *P. tricornutum* pPtPuc1 and pPtPuc3 colonies, respectively. No episomes could be rescued from *P. tricornutum* pPtPuc2 and pPtPuc4 exconjugants; phleomycin-resistant colonies obtained with these plasmids were likely the result of chromosomal integration. This result was consistent with the 1.4-kb *CEN6-ARSH4-HIS3* fragment (present on plasmids pPtPuc1 and pPtPuc3) containing a sequence establishing episomal replication in *P. tricornutum*. When rescued episomes from pPtPuc1 and pPtPuc3 were analysed by gel electrophoresis, 8/20 and 10/20, respectively, had sizes identical to the original material used to conjugate *P. tricornutum*. Thus, the originally isolated *P. tricornutum* sequence found in p0521s (that is, R1 and R2) was not essential for episomal replication in *P. tricornutum*, and the vector-derived sequence, *CEN6-ARSH4-HIS3*, was sufficient to establish replicating episomes. Determining how the *CEN6-ARSH4-HIS3* fragment confers episomal replication is the subject of ongoing research.

### Characteristics of episome replication in diatoms

**Figure 2:**
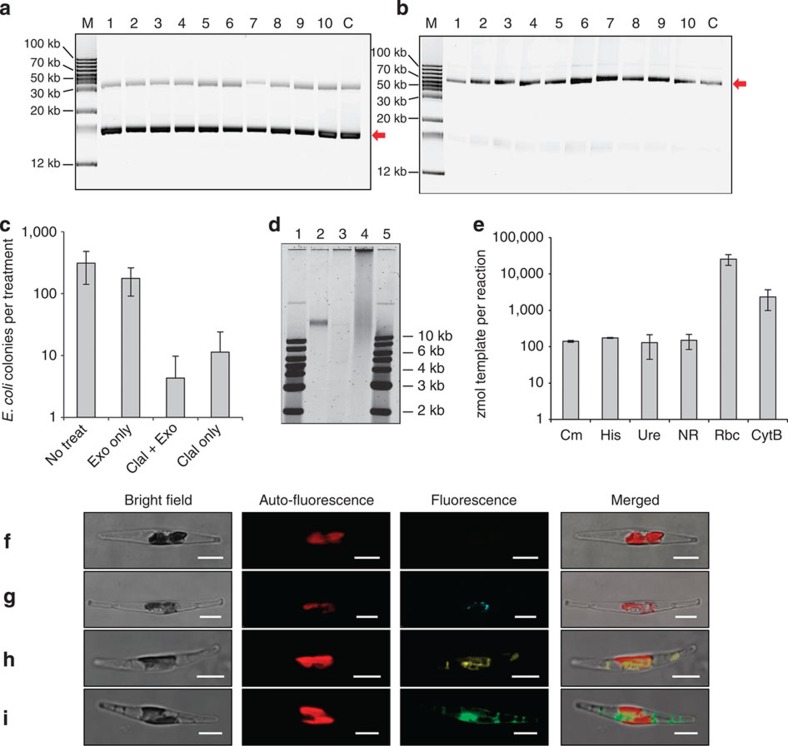
Demonstration that *P. tricornutum* episomes replicate as stable, circular, low-copy plasmids (**a**–**e**), and expression and localization of proteins encoded on the *P. tricornutum* episome p0521s (F-I). (**a**) Cultures of *P. tricornutum* containing p0521s (clone 9, Supplementary Fig. 3), were subcultured in seawater medium for 28 days with or without antibiotic selection and plated (Supplementary Fig. 5). DNA from five antibiotic-resistant colonies from each culture (Supplementary Fig. 5D) was recovered in *E. coli* and isolated plasmids were separated by agarose gel electrophoresis. Shown are rescued plasmids derived from separate *P. tricornutum* colonies that were initially subcultured for 28 days without (lanes 1–5) or with (lanes 6–10) antibiotic selection. ‘M' designates supercoiled marker^41^ and ‘C' designates the original plasmid (isolated from clone 9) introduced into *P. tricornutum*. Arrow denotes supercoiled plasmid band. (**b**) Stability of p0521-Se containing a 49-kb *S. elongatus* fragment. Ten independently transformed *P. tricornutum* lines containing p0521-Se were subcultured in liquid media with selection for 60 days, followed by episome rescue and separation of plasmids by agarose gel electrophoresis. Arrow denotes supercoiled plasmid band. (**c**) Plasmids extracted from *P. tricornutum* were untreated, treated with exonuclease, ClaI endonuclease or a combination of exonuclease and ClaI. Treated plasmids were transformed into *E. coli* and the number of transformed colonies was plotted (error bars indicate one s.d. of the mean from three biological replicates). (**d**) Agarose gel electrophoresis of plasmids extracted from *P. tricornutum* and treated with nucleases. Lanes from left to right: (1) 1 kb^+^ ladder (NEB), (2) p0521s control (from *E. coli*), (3) p0521s exonuclease-treated (extracted from *P. tricornutum*), (4) p0521s untreated (extracted from *P. tricornutum*), (5) 1 kb^+^ ladder (NEB), (**e**) Copy number of p0521s in *P. tricornutum* determined by qPCR. Cm (*Cat* gene) and His (*HIS3* gene) are loci found on the episome backbone; Ure (urease, protein ID 29702) and NR (nitrate reductase, protein ID 54983) are loci encoded on *P. tricornutum* nuclear chromosomes 18 and 20, respectively; Rbc (RuBisCO small subunit) and CytB (Cytochromoe B) are loci found on the *P. tricornutum* chloroplast and mitochondrial chromosomes, respectively. Error bars denote one s.d. of the mean from three biological replicates. (**f**) Wild-type *P. tricornutum*, fluorescence measured with GFP settings. (**g**) *P. tricornutum* expressing CFP translationally fused to beta-carbonic anhydrase (Protein ID 51305) localized to the chloroplast pyrenoid encoded on plasmid p0521s. (**h**) *P. tricornutum* expressing YFP translationally fused to mitochondrial urea transporter (Protein ID 39772) encoded on plasmid p0521s. (**i**) *P. tricornutum* expressing GFP localized to the cytoplasm encoded on plasmid p0521s. Scale bar for **f**–**i** indicates 5 μm.

Once the episomes were established in the diatom cell, they were maintained with high fidelity. In the absence of selection after an estimated 30 generations, an average of 35% of the cells retained the p0521s plasmid corresponding to a segregation efficiency of 97% (Fig. 2a, Supplementary Table 4, Supplementary Fig. 5). We were also successful in maintaining a 49-kb heterologous sequence from the cyanobacterium *Synechococcus elongatus* on the episome at the correct size (p0521-Se, Fig. 2b) for two months in the presence of the antibiotic zeocin. Episomes (p0521s and p0521) were maintained in *P. tricornutum* in closed circular form as determined by exonuclease treatment on extracted *P. tricornutum* DNA before episome rescue (Fig. 2c,d, Supplementary Fig. 6). Southern blot analysis in which DNA was extracted from *P. tricornutum* p0521s-containing lines also supported circular replication and absence of genomic integration (Supplementary Fig. 7). Finally, quantitative PCR (qPCR) assays supported maintenance of the episome at native chromosomal copy number in *P. tricornutum* (Fig. 2e, Supplementary Fig. 6). The biotechnological utility of the diatom episome was tested by expressing multiple fusions of fluorescent proteins with proteins of known localization^23^,^25^,^26^ (Fig. 2f–i). Altogether, the data is consistent with p0521s maintained as a stable, circular, episome replicating at a similar copy number as native nuclear *P. tricornutum* chromosomes, allowing for efficient protein expression.

### Extension of methods to a centric diatom

**Figure 3:**
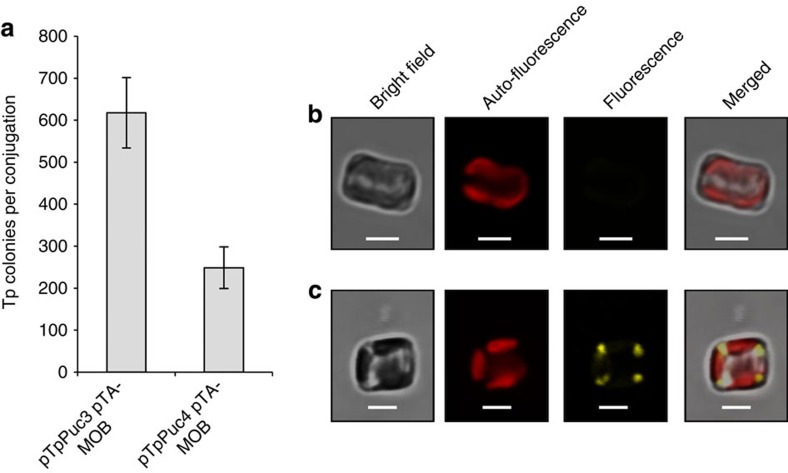
Episome replication in *T. pseudonana*. (**a**) Conjugation from *E. coli* to *T. pseudonana* results in increased conjugation efficiency when the yeast *CEN6-ARSH4-HIS3* region is included on the plasmid (pTpPuc3) compared with control plasmid lacking this region (pTpPuc4). Error bars denote one s.d. of the mean from at least three biological replicates. (**b**,**c**) Images of *T. pseudonana* wild type (**b**) and exconjugants expressing YFP translationally fused to PEPCK (Protein ID_5186) encoded on a p0521s-derived episome (**c**). Scale bar, 2.5 μm.

We further investigated if our system for genetic manipulation of *P. tricornutum* could be transposed to the centric diatom *T. pseudonana* that has fully silicified shell. Episomes were modified to have the nourseothricin antibiotic selectable marker cassette that functions in *T. pseudonana*. Using a variant of plasmid pPtPuc3 that we called pTpPuc3, we successfully conjugated the episome into *T. pseudonana* with an efficiency of 2.0 × 10^−4^ diatom cells (Fig. 3). While plasmids with the *CEN6-ARSH4-HIS3* region (pTpPuc3) or without (pTpPuc4) both yielded *T. pseudonana* colonies, only the pTpPuc3 episome could be successfully rescued consistent with the yeast sequence conferring episomal replication ability in *T. pseudonana*. Plasmids recovered after episome rescue from *T. pseudonana* were identical in size to the original plasmid control (Supplementary Fig. 8 and Supplementary Table 3). We constructed a version of p0521s that had the nourseothricin resistance gene driven by a *T. pseudonana* promoter and that also encoded a translational fusion between yellow fluorescent protein (YFP) and *T. pseudonana* phosphoenolpyruvate carboxykinase (PEPCK). Introduction of this plasmid resulted in *T. pseudonana* exconjugants with highly visible YFP signal in the mitochondria as expected^27^,^28^ (Fig. 3). Altogether the results observed for *T. pseudonana* mirror the findings in *P. tricornutum* and established that the episome/conjugation system operates in a similar fashion in both diatom species.

## Discussion

**Figure 4:**
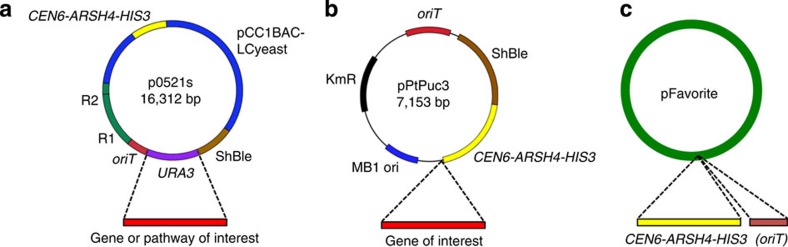
Suggested strategies to clone sequences of interest into diatom episomes. (**a**) Genes or pathways of interest can be assembled into p0521s replacing the *URA3* gene. Incoming DNA should be prepared with sequence overlaps to the plasmid regions flanking *URA3* and assembled in yeast spheroplasts with counter-selection on 5FOA. (**b**) Smaller sequences interest (equivalent in size to single expression cassettes) can be assembled into pPtPuc3 using Gibson assembly or other cloning strategy. Sequences can be introduced anywhere in the plasmid, but we typically insert at the 3′ region of the *CEN6-ARSH4-HIS3* sequence. (**c**) Any existing plasmid can be modified with the yeast *CEN6-ARSH4-HIS3* sequence amplified from p0521s, pPtPuc3 or other source to enable episomal replication in *P. tricornutum* or *T. pseudonana*. The *oriT* sequence should also be included (if it is not already present in the vector sequence) if conjugation from *E. coli* is chosen as the method of plasmid introduction.

We discovered a small sequence (1.4 kb) from yeast that permits low-copy episomal replication in the diatoms *P. tricornutum* and *T. pseudonana*. This small yeast sequence (*CEN6-ARSH4-HIS3)* contains the plasmid maintenance functions for yeast centromeric plasmids (*CEN6* and *ARSH4)* and the *HIS3* gene to complement yeast histidine auxotrophy. We constructed two replicating plasmids for *P. tricornutum*, p0521s and pPtPuc3, and each contains the *CEN6-ARSH4-HIS3* sequence. A plasmid identical to pPtPuc3, but with the antibiotic selection replaced with the *T. pseudonana* nourseothricin resistance cassette was also constructed (pTpPuc3). Plasmid p0521s has an ShBle cassette for *P. tricornutum* selection with phleomycin as well as the pCC1BAC backbone to support large inserts (up to several hundred kilobases) in *E. coli*. Cloning sequences of interest into this backbone can be performed using yeast assembly methods^29^,^30^ and inserts can be designed to replace the *URA3* gene with counter selection on 5-fluorooritic acid (5FOA; Fig. 4). When cloning genes of interest into p0521s using yeast assembly, predigestion of the plasmid with I-CeuI and I-SceI is recommended to increase the recombination efficiency. For expression of smaller sequences in *P. tricornutum* (for example, single-gene expression cassettes), pPtPuc3 is better suited with a smaller backbone that can be amplified by PCR and assembled with sequences of interest using Gibson assembly^31^. Note that the high copy number of pUC19-based plasmids may complicate cloning sequences larger than ∼10 kb. To clone sequences into pPtPuc3, we typically insert sequences of interest immediately following the 3′ end of the *CEN6-ARSH4-HIS3* region. Plasmid pTpPuc3 can be engineered with genes in a similar manner as described above for pPtPuc3. All three plasmids (p0521s, pPtPuc3 and pTpPuc3) are available at Addgene. Alternatively, the *CEN6-ARSH4-HIS3* region can simply be amplified and added to any plasmid to permit replication as an episome, provided a means of selection for transformed cells is also present on the plasmid (Fig. 4). Further inclusion of an *oriT* is necessary if introduction by conjugation is also desired.

While our initial approach was to discover diatom DNA sequences that function as centromeres, the resulting finding that a yeast-derived sequence could function in a similar manner was surprising and could help elucidate native diatom centromere sequences and sequence requirements in ongoing research. Whether the yeast-derived sequence functions as a true centromere in diatoms remains to be tested. Although maintenance of the *P. tricornutum* episome without antibiotic selection was not as high as the ∼80–95% retention observed for yeast centromeric vectors^18^, it is higher than yeast vectors based on replication origins (ARS) in which only 1% of cells maintain the plasmid in the absence of selection after an equivalent number of generations^19^. Furthermore, as noted for yeast^29^, most of the rearrangements of the diatom episome appear to occur during plasmid introduction; once a line was verified to have the correctly sized plasmid in *P. tricornutum*, the episome replicated without alteration over long periods of time (Fig. 2a, Supplementary Fig. 5). Interestingly, the *CEN6* (13% GC) and *ARSH4* (30% GC) regions have much lower GC content relative to the average for *P. tricornutum* (48% GC). Low GC regions are known key features in red algal and other protist centromeres^22^,^32^ and may be functioning in a similar manner in *P. tricornutum*.

The diatom system described above is the second system (apart from yeasts^13^,^16^,^33^) in which an episome was introduced into a eukaryotic cell by conjugation and stably maintained. Conjugative delivery of such elements results in order of magnitude gains in efficiency over widely used particle bombardment methods^6^, both in time and materials, at the genetic manipulation and screening stages. In addition, a large DNA sequence (49 kb) was successfully introduced and maintained in *P. tricornutum* using this system, suggesting that an entire metabolic pathway could be introduced into the diatom, which may facilitate and accelerate biotechnological applications in these organisms. We observed great flexibility in the conditions that favour conjugation (Supplementary Table 2), but presented the optimum protocols that we have developed. For *P. tricornutum*, we found that diatom cells grown on agar plates were transformed by conjugation at a higher rate than liquid grown cells, while *T. pseudonana* cells were best grown in liquid culture due to difficulties growing this diatom on plates for extended periods. In adapting the technique to other diatom species, plasmids should minimally have the *CEN6-ARSH4-HIS3* region, an origin of transfer (*oriT*) and a selectable marker functioning in the diatom of interest. Conditions during the conjugation (60–90 min at 30 **°**C on ½ strength L1 agar +5% LB medium) are a compromise between those optimal for *E. coli* (LB agar, 37 **°**C) and those optimal for the diatom (L1 medium, 18 **°**C) and will likely require empirical determination for other diatom species.

In addition to its biotechnological utility, bacterial conjugation to diatoms is interesting in light of the complex evolutionary history of the Stramenopile clade, which is characterized by multiple endosymbiotic events^34^ and recent horizontal gene transfers from marine bacteria^35^,^36^. Diatom genomes contain a high percentage of recently transferred bacterial genes, and transkingdom conjugation may provide a possible mechanism to explain the acquisition of some of the bacterial DNA that makes up 5–10% of diatom genomes^36^. The results also add credence to the increasing awareness that conjugation influences microbial ecology in the oceans^37^,^38^,^39^.

The ease, simplicity and scalable nature of these novel tools makes them amenable to an efficient and high-throughput functional genetic system for diatoms. This system represents a new and exciting development to begin to make rapid and fundamental advances in gene function and to start understanding the molecular regulation controlling the biology of these globally significant marine phytoplankton species.

## Methods

### Microbial strains and growth conditions

*Saccharomyces cerevisiae* VL6-48 (ATCC MYA-3666: *MATα his3*-Δ*200 trp1*-Δ*1 ura3-52 lys2 ade2-1 met14 cir*^*0*^) cells were grown in rich medium (YEPD) or complete minimal medium lacking histidine or histidine and uracil or histidine to which 1 g l^−1^ of 5-fluoroorotic acid (5FOA) was added (Teknova). In addition, 60 mg l^−1^ adenine sulfate was added to all yeast media. All complete minimal media used for plating yeast cells directly after spheroplast transformation contained 1 M Sorbitol.

*Escherichia coli* (Epi300, Epicentre) were grown on Luria broth or agar supplemented with chloramphenicol (20 mg l^−1^) or kanamycin (50 mg l^−1^) or ampicillin (50 mg l^−1^) or tetracycline (10 mg l^−1^) or gentamicin (20 mg l^−1^) or combinations of these as needed.

*E. coli* strains containing plasmids p0521s (GenBank accession KP745602), pPtPuc3 (GenBank accession KP745601) and pTpPuc3 (GenBank accession KP745603) described in this manuscript have been deposited at Addgene.com and are available for request.

*Phaeodactylum tricornutum* was grown in L1 medium at 18 **°**C under cool white fluorescent lights (50 μE m^−2^ s^−1^).

*L1 medium*. For liquid medium combine: 1 l Aquil Salts (Synthetic Seawater), 2 ml NP stock, 1 ml L1 trace metals stock, 0.5 ml f/2 vitamin solution. Filter sterilize through a 0.2-μm filter. For agar plates, combine one part sterilized liquid L1 medium and one part autoclaved 2% Bacto agar and pour into petri dishes.

*Aquil salts*. (Two separate solutions, anhydrous and hydrous salts, are made at 2 × strength and mixed to make Aquil salts).

*Anhydrous salts*. Anhydrous salts (resuspend in 500 ml): NaCl 24.5 g, Na_2_SO_4_ 4.09 g, KCl 0.7 g, NaHCO_3_ 0.2 g, KBr 0.1 g, H_3_BO_3_ 0.03 g or 3 ml 10 mg ml^−1^ stock, NaF 0.003 g or 300ul 10 mg ml^−1^ stock.

*Hydrous salts*. Hydrous salts (resuspend in 500 ml): MgCl_2_ 6 H_2_0 11.1 g, CaCl_2 2_H_2_0 1.54 g.

*NP stock*. NaNO_3_ 37.5 g per 100 ml, NaH_2_PO_4_-H_2_0 2.5 g per 100 ml.

*L1 trace metals*. Mix-up the following to make 1 l at 1,000 × (stock solutions in parentheses): FeCl_3_·6H_2_O 3.15 g, Na_2_EDTA·2H_2_O 4.36 g, CuSO_4_·5H_2_O (9.8 g l^−1^ dH_2_O) 0.25 ml, Na_2_MoO_4_·2H_2_O (6.3 g l^−1^ dH_2_O) 3.0 ml, ZnSO_4_·7H_2_O (22.0 g l^−1^ dH_2_O) 1.0 ml, CoCl_2_·6H_2_O (10.0 g l^−1^ dH_2_O) 1.0 ml, MnCl_2_·4H_2_O (180.0 g l^−1^ dH_2_O) 1.0 ml, H_2_SeO_3_ (1.3 g l^−1^ dH_2_O) 1.0 ml, NiSO_4_·6H_2_O (2.7 g l^−1^ dH_2_O) 1.0 ml, Na_3_VO_4_ (1.84 g l^−1^ dH_2_O) 1.0 ml, K_2_CrO_4_ (1.94 g l^−1^ dH_2_O) 1.0 ml.

*F/2 vitamin solution*. Thiamine-HCl add 200 mg powder l^−1^, Biotin add 10 ml l^−1^ of a 0.1 g l^−1^ stock, Cyanocobalamin add 1 ml l^−1^ of a 1 g l^−1^ stock.

*Thalassiosira pseudonana* (Hustedt) Hasle et Heimdal (clone CCMP 1335) from the Provasoli Guillard NCMA (National Center for Marine Algae and microbiota, Maine, USA) were grown between 18 and 22 **°**C, using 0.2 μm filtered and boiled nutrient-poor seawater (Scripps pier, La Jolla, California: lat-long N 32.86671 and W 117.25587, collected on 4 April 2014) with metals and vitamins added to achieve f/2 trace element concentrations^40^. Phosphate was supplied as 25 μM, silicate was provided at 100 μM (Na_2_SiO_3_ 9H_2_O) and nitrogen was supplied as a mixture of 200 μM ammonium chloride (NH_4_Cl) plus 200 μM sodium nitrate (NaNO_3_). Cultures were illuminated on a light/dark cycle of 16/8 h with cool white fluorescent lamps (Vita-Lite 5500 K, DUROTEST, USA) at photon flux densities between 80 and 130 μE m^−2^ s^−1^. *T. pseudonana* cell plating was conducting using three-fourth of the seawater media described above, plus one-fourth double distilled water and 8 g l^−1^ of bactoagar (Sigma).

### DNA isolation

Plasmid DNA was isolated using the modified alkaline lysis protocol described below. Steps 1–3 are variable depending on the species, while steps 4–10 are common for all species.

*Steps 1–3 for S. cerevisiae*. (1) Yeast culture (5–10 ml) was grown to high density. (2) Next yeast cells were pelleted at 3,000*g* for 5 min and supernatant was discarded. (3) Cells were resuspended in 250 μl resuspension buffer, which contained 240 μl P1 (Qiagen), 5 μl of 1.4 M β-Mercaptoethanol and 5 μl Zymolyase solution (Zymolyase solution: 200 mg Zymolyase 20 T (USB), 9 ml H_2_O, 1 ml 1 M Tris pH7.5, 10 ml 50% glycerol, stored at −20 °C) and incubated at 37 **°**C for 60 min.

*Steps 1–3 for P. tricornutum and T. pseudonana*. (1) Cultures (10–20 ml) were harvested during exponential growth phase. (2) Cells were pelleted at 4,000*g* for 5 min, supernatant was discarded. (3) Cells were resuspended in 250 μl resuspension buffer, which contained 235 μl P1 (Qiagen), 5 μl hemicellulase 100 mg ml^−1^, 5 μl of lysozyme 25 mg ml^−1^, and 5 μl Zymolyase solution (Zymolyase solution: 200 mg Zymolyase 20T (USB), 9 ml H_2_O, 1 ml 1 M Tris pH7.5, 10 ml 50% glycerol, stored at −20 °C) and then cells were incubated at 37 °C for 30 min.

*Steps 1–3 for E. coli*. (1) Two ml overnight cultures were used to inoculate 25 ml LB median containing appropriate antibiotic and induction solution (Epicentre) and grown for 4–5 h in 37 °C shaker. (2) Next *E. coli* cells were pelleted at 4,000*g* for 5 min, supernatant was discarded. (3) Cells were resuspended in 250 μl.

*Steps 4–10 common for all species*. (4) Two hundred and fifty μl lysis buffer P2 (Qiagen) was added and samples were inverted 5–10 times to mix. (5) Then 250 μl of Neutralization buffer P3 was added and samples were inverted 5–10 times to mix. (6) Then samples were spun down at 16,000*g*, 10 min. (7) Supernatant was transferred to a clean tube and 750 μl isopropanol was added and the samples were mixed by inversion and spun down at 16,000*g*, 10 min. (8) Next the supernatant was removed and 750 μl 70% EtOH was added and samples were mixed by inversion and span down at 16,000*g*, 5 min. (9) Next the supernatant was discarded and pellets were resuspended in 50–100 μl of TE buffer. (10) After that the samples were kept at 37 °C for 30–60 min to dissolve.

### Transfer of DNA into P. *tricornutum* by electroporation

Culture (200 ml) in exponential phase (bulk florescence=50) was centrifuged at 3,000*g* for 5 min. Supernatant was removed, cells were resuspended in 1 ml of 0.5 M NaCl, 50 mM mannitol and centrifuged at 3,000*g* for 5 min. Next the supernatant was removed and cells were resuspended in 1 ml of 1 M Sorbitol. Next 400 μl of cells were removed and mixed with 10 μl plasmid DNA (∼ 2 μg μl^−1^). The mixture was moved to a 0.2-cm electroporation cuvette (Bio-Rad) and subjected to electroporation at 700 V, 200 Ω, 25 μF. Then the cells were resuspended in 20 ml of L1 media and grown for 2 days before transfer to selection plates containing phleomycin (20 μg ml^−1^).

### PEG DNA transformation method

*P. tricornutum* cells were grown in L1 liquid medium or on plates. Liquid cultures were spun for 5 min at 4,000*g* at 10 °C. Supernatant was removed and the cells were counted using a haemocytometer. Next the cells were resuspended to give a final concentration in the range of 3–6 × 10^8^ cells ml^−1^. For *P. tricornutum* grown on plates, 250 μl of *P. tricornutum* culture adjusted to 1.0 × 10^8^ cells ml^−1^ was plated on ½ L1, 1% agar plates and grown for 4 days, then 500 μl of L1 media was added to the plate and cells were scraped to collect. Then cell concentration was adjusted to 3–6 × 10^8^ cells ml^−1^. Next 1 ml of cells was resuspended in 9 ml of filter sterilized spheroplasting solution (20 μl of Zymolase 100T (10 mg ml^−1^), 100 μl of freshly made lysozyme (25 mg ml^−1^), 0.1 g of hemicellulase and L1 solution was added to final volume of 9 ml) and incubated for 30 min at 37 °C. Next 40 ml of L1 solution was added and mixed by inverting, followed by centrifugation for 5 min at 3,000*g*, 10 °C. Then supernatant was removed and cells were respuspened in 500 μl of L1 media. Spheroplasts (250 μl) were transferred to 1.5 ml Eppendorf tube, and 25 μl of DNA (∼1 μg μl^−1^) was added and immediately 1 ml of 25% PEG 8,000, 10 mM Tris pH 8, 10 mM CaCl_2_, 2.5 mM MgCl_2_, pH 8 equilibrated at 37 °C was added and tubes were inverted four to six times. Then the mixture was incubated at room temperature for 10 min. Next the mixture was centrifuged for 7 min at 1,500*g*. Supernatant was removed and cells were resuspended in 30 ml of L1 and incubated for 45 min without selection at 18 °C. Then the cells were centrifuged for 5 min at 3,000 r.p.m., 15 °C. Then the cells were resuspended in 600 μl of L1 and 200 μl was plated on ½ × L1, 20 μg ml^−1^ chloroamphenicol. After 2 days, the cells were scraped (in 500 μl of L1 media) and plated on 0.5 × L1, 1% agar plates containing 20 μg ml^−1^ phleomycin. Colonies appeared after 10–14 days.

### Transfer of DNA to P. *tricornutum* via conjugation from *E. coli*

*Preparation of P. tricornutum cells*. Two hundred and fifty μl of liquid grown culture adjusted to 1.0 × 10^8^ cells ml^−1^ was plated on ½L1, 1% agar plates and grown for 4 days. Then 500 μl of L1 media was added to the plate and cells were scraped and counted using a hemocytometer. Next the concentration was adjusted to 5 × 10^8^ cells ml^−1^.

*Preparation of E. coli cells*. Culture (50 ml) was grown at 37 **°**C to OD_600_ of 0.8–1.0 then spun down for 10 min at 3,000*g* and resuspended in 500 μl of SOC media.

*Conjugation of P. tricornutum with E. coli*. *P. tricornutum* cells (200 μl) was moved to a 1.5-ml microfuge tube and then 200 μl of *E. coli* cells were added and mixed by pipetting up and down few times. Next the cells were plated on ½ × L1, 5% LB, 1% agar plates and incubated for 90 minutes at 30 **°**C in the dark, then moved to 18 **°**C in the light and grown for 2 days. After 2 days, 1 ml of L1 media was added to plates and cells were scraped. Two hundred μl of the scraped cells was plated on ½ × L1, phleomycin 20 μg ml^−1^, 1% agar plate and incubated at 18 **°**C in the light. Colonies appeared after 10–14 days.

### Transfer of DNA to T. *pseudonana* via conjugation from *E. coli*

*Preparation of T. pseudonana cells*. Five hundred ml of liquid grown culture were spun for 5 min at 4,000*g* at 10 **°**C, then most of the media were removed, cells were counted using a haemocytometer and the concentration was adjusted to 2 × 10^8^ cells ml^−1^.

*Preparation of E. coli cells*. Culture (150 ml) grown at 37 **°**C to OD_600_ of 0.3 was spun down for 10 min at 3,000*g* and resuspended in 800 μl of SOC media.

*Conjugation of T. pseudonana with E. coli*. *T. pseudonana* cells (200 μl) was moved to 1.5 ml microfuge tube and then 200 μl of *E. coli* cells were added and mixed by pipetting up and down a few times. Next the cells were plated on ½ × *T. pseudonana* medium, 5% LB, 1% agar plates and incubated for 90 min at 30 **°**C in the dark, then moved to 18 **°**C in the light and grown for 4 h. Then 1 ml of *T. pseudonana* medium was added to the plate and cells were scraped. Two hundred μl of the scraped cells was plated on ½ × *T. pseudonana* medium, Nourseothricin 50 μg ml^−1^, 1% agar plate and incubated at 18 **°**C in the light. Colonies appeared after 7–14 days.

### Isolation and modification of p0521o

Plasmids containing one of each of the five fragments were introduced into *P. tricornutum*. Plasmids containing what we originally thought to be fragment 5 from scaffold 25 were later found to be a concatenation of fragments 1 and 5 after Ion Torrent Sequencing (Supplementary Fig. 1b). Plasmids recovered from *P. tricornutum* contained either fragment 1 (for example, p0319 or p0521o) or a reduction of fragment 5 (for example, p0524_3 and p0524_4). A plasmid containing fragment 4 was unexpectedly obtained by an unknown mechanism (p0413; Supplementary Fig. 1c). After reintroduction into *P. tricornutum*, plasmid p0521o was recovered at its identical size while plasmid p0524_4 was further reduced (Supplementary Fig. 1d). After extended maintenance of plasmid p0521o, a spontaneously minimized form was eventually isolated, which we called p0521o-reduced. To facilitate further molecular biology with these plasmids, p0521 and p0521s were created from plasmids p0521o and p0521o-reduced using the following procedure. Plasmids p0521o and p0521o-reduced were transformed into yeast using yeast spheroplast transformation and were modified with the *URA3* cassette as follows. The *URA3* cassette was amplified by PCR with flanking I-SceI and I-CeuI sites and homology added to the ShBle and *oriT* sequences using primers 0521URA_F and 0521URA_R (Supplementary Table 1). This PCR product was transformed into yeast cells carrying plasmids p0521o and p0521o-reduced using the lithium acetate method to insert the *URA3* cassette between the ShBle and the *oriT* regions generating plasmids p0521 and p0521s (Fig. 1a).

### Other plasmid construction

Vectors for cloning each of the large DNA fragments from *P. tricornutum* chromosome 25 were assembled in yeast by co-transforming XhoI-digested template plasmid pBK-RBYV (Supplementary Fig. 9) and two 200-bp PCR-amplified *P. tricornutum* DNA homology regions corresponding to the two ends of the fragment to be cloned. Each of the 200-bp PCR products contained 25–40 bp homologies to the vector pBK-RBYV. Primers to create these 200-bp sequences to TAR clone fragments F1 through F5 can be found in Supplementary Table 1. Once the cloning vectors were assembled, they were digested with XhoI to open the vector and excise the *URA3* cassette allowing for recombination with the digested *P. tricornutum* DNA.

TAR cloning of *P. tricornutum* fragments was based on previously developed protocols^41^. *P. tricornutum* DNA was first isolated in agarose plugs using the Bio-Rad CHEF Genomic DNA Plug Kit. To prepare the plugs, 50 ml of mid-log phase (10^8^ cells ml^−1^) *P. tricornutum* culture was centrifuged at 1,500*g* for 5 min at 10 **°**C. Cells were washed once with 50 ml of 1 M sorbitol and were resuspended in 2 ml of SPEM solution (1 M sorbitol, 10 mM EDTA pH 7.5, Na_2_HPO_4_ 7H_2_O (2.08 g l^−1^), NaH_2_PO_4_·1H_2_O (0.32 g l^−1^)). The cell suspension was incubated for 5 min at 37 **°**C and mixed with an equal volume of 2.0% low-melting point agarose in 1 × TAE buffer (40 mM Tris, 20 mM acetic acid and 1 mM EDTA), which was equilibrated at 50 **°**C. Aliquots of 100 μl were transferred into plug moulds (Bio-Rad, catalogue #170–3713) and allowed to solidify for 10 min at 4 **°**C. Next the plugs were removed from the moulds into a 50-ml conical tube containing 5 ml of protoplasting solution (4.56 ml of SPEM solution, 200 μL Zymolyase-100T solution (50 mg ml^−1^ dissolved in H_2_O), 200 μl lysozyme (25 mg ml^−1^), 40 μl β-Mercaptoethanol and incubated for 1 h at 37 **°**C. Next the plugs were washed with 25 ml of wash buffer (20 mM Tris, 50 mM EDTA, pH 8.0) and then incubated in 5 ml in Proteinase K buffer (100 mM EDTA (pH 8.0), 0.2% sodium deoxycholate and 1% sodium lauryl sarcosine, 1 mg ml^−1^ Proteinase K) for 24 h at 50 **°**C. After proteinase K treatment, plugs were washed four times in wash buffer and stored at 4 **°**C.

Agarose plugs containing *P. tricornutum* DNA were digested with AscI, washed in TE buffer (pH 8) and melted in 100 μl TE buffer at 65 **°**C for 10 min. The molten agarose was then equilibrated at 42 **°**C for 10 min and 2 μl of β-agarase (NEB) was added and incubated for 8 h. Ten μl of DNA from plugs, (typically 100 ng μl^−1^) was mixed with 300–500 ng of vector and the assembly was performed by mixing the DNA with yeast spheroplasts^42^ and plating on complete minimal medium lacking histidine.

Plasmids based on p0521s were constructed using yeast assembly as described above for TAR cloning of *P. tricornutum* fragments F1 through F5 (ref. 41). For example, a 48,602-bp region from *Synechococcus elongatus* PCC 7942 (chromosome coordinates 223,718 to 272,317) was TAR cloned from previously cloned BAC DNA (previously cloned as fragment 3 (ref. 29)) after digest of BAC DNA with the restriction enzyme FseI. A cloning vector with 200-bp homology regions was prepared as described above (see Supplementary Table 1 for primer sequences). Three regions of the *S. elongatus* insert were checked by multiplex PCR (see Supplementary Table 1 for sequences) and plasmids were analysed by agarose gel electrophoresis to confirm that they had the correct size.

The pPtPuc plasmids to test the relative importance of the *P. tricornutum*-derived sequences and yeast-derived sequences were constructed as follows. First, a version of pUC19 was constructed containing the transfer origin (*oriT*) from pRL2948a (originally derived from conjugative plasmid RK2, C. P. Wolk, unpublished data). This region was PCR amplified with primers Puc-oriT-1 and Puc-oriT-2 and cloned into NdeI and SfoI sites of pUC19 within the *lacZ* alpha region using Gibson assembly to make pUC-oriT. Next, plasmid pUC-oriT was modified with the kanamycin-resistance cassette inserted into the ampicillin cassette to make plasmid pUC-oriT-Km. The kanamycin-resistance cassette was amplified from pACYC177 using primers Puc-oriT-km1+Puc-oriT-km2 in the ScaI-digested pUC-oriT plasmid. To make plasmids pPtPuc1 through pPtPuc4, plasmid pUC-oriT-Km was digested with EcoRI and BamHI at the multiple cloning site and assembled with inserts as described below. Templates for PCR amplification of inserts include pAF6 (ref. 43) for PtShBle, p0521s for the *P. tricornutum* region and pCC1BAC-LCYEAST^44^ for the *CEN6-ARSH4-HIS3* region.

For pPtPuc1, PtShBle was amplified with primers PtPuc1a+PtPuc12e, the *P. tricornutum* region was amplified with primers PtPuc2b+PtPuc13f and the CEN-ARS-HIS region was amplified with primers PtPuc3c+PtPuc14d. For pPtPuc2, PtShBle was amplified with primers PtPuc1a+PtPuc12e, and the *P. tricornutum* region was amplified with primers PtPuc2b+PtPuc5d. For pPtPuc3, PtShBle was amplified with primers PtPuc1a+PtPuc15f, and the *CEN6-ARSH4-HIS3* region was amplified with primers PtPuc6c+PtPuc14d. For pPtPuc4, PtShBle was amplified with primers PtPuc1a+PtPuc7d.

A fifth plasmid, pPtPuc7, was constructed containing the ShBle coding region regulated by *Cylindrotheca fusiformis* promoter and terminator (CfShBle)^45^ and the *CEN6-ARSH4-HIS3*. Thus, this plasmid contained no *P. tricornutum* sequence and was assembled by amplifying the CfShBle with primers PtPuc8a+PtPuc17f and the *CEN6-ARSH4-HIS3* region with primers PtPuc10c+PtPuc14d.

To make the fluorescent protein translational fusion expression vectors, expression cassettes consisting of a promoter, a fluorescent protein by itself or fused in frame to a protein of known localization and a terminator were first assembled in pUC19 vectors. For pXFP11, the *P. tricornutum* nitrate reductase promoter (Protein ID 54983) was amplified from *P. tricornutum* genomic DNA using primers NewXFP1+NewXFP12, the green fluorescent protein (GFP) gene was amplified using primers NewXFP13+NewXFP14, and nitrate reductase terminator was amplified from *P. tricornutum* genomic DNA using primers NewXFP15+NewXFP16. These three PCR products were purified using the QIAquick PCR purification kit (Qiagen) and assembled into an EcoRI- and HindIII-digested pUC19 plasmid using Gibson Assembly. The cassette was then reamplified with primers YeastXFPF4+YeastXFPR2 and assembled into p0521s using yeast assembly.

To make a mitochondrion-localized fluorescent protein fusion expression cassette, the mitochondrial urea transporter (Protein ID 39772) was amplified from *P. tricornutum* genomic DNA using primers NewXFP11+ HA-MTUT-YFP-Term-4 omitting the stop codon. YFP was amplified using primers Fcp-MtUT-YFP-Term-5+HA-MtUT-YFP-Term-6. Regulation for this cassette was provided by the FcpB promoter amplified from *P. tricornutum* using primers NewXFP9+NewXFP10 and the FcpA terminator using primers HA-MtUT-YFP-Term-7+HA-MTUT-YFP-Term-8. These four PCR products were purified using the QIAquick PCR purification kit (Qiagen) and assembled into an EcoRI- and HindIII-digested pUC19 plasmid using Gibson Assembly. The cassette was then amplified using primers YeastXFPF5+YeastXFPR1 and assembled into p0521s using yeast assembly.

To make pXFP3, the cassette consisting of FcpB promoter, the β-carboxyanhydrase open reading frame (Pt protein #51305) that was translationally fused to cyan fluorescent protein (CFP) and the FcpA terminator was amplified from a previously constructed template plasmid^26^ using primers YeastXFPF2+YeastXFPR1 and assembled into p0521s using yeast assembly.

The PEPCK from *T. pseudonana* (protein ID#5186) was cloned from complementary DNA using the primers Tp-PEPCK-F and Tp-PEPCK-R (Supplementary Table 1), replacing the stop codon with TTA-leucine. The PCR product was then introduced into pENTR/D/Topo (Invitrogen) and sequenced. PEPCK was then transferred into a custom-built Gateway-compatible destination vector for YFP fusion in carboxyl-terminal (pTpDEST-C′YFP) by LR recombination according to the manufacturer's instructions (Invitrogen). Details of pTpDESTC′YFP construction will be described in a forthcoming publication. The plasmid, pTpExpPEPCK-YFP, contained the *T. pseudonana*-specific nourseothricin resistance marker flanked by the LHCF9 promoter (983-nt upstream of the *T. pseudonana* protein ID 268127) and the LHCF9 terminator (503-nt downstream of the *T. pseudonana* protein ID 268127) amplified from pTpfcp/nat^5^ followed by the expression cassette composed of the PEPCK-YFP fusion flanked by the LHCF9 promoter and terminator. The junction between PEPCK and YFP was checked by sequencing to ensure in frame cloning. Finally, the 6,377-bp region containing the resistance cassette and the expression cassette was reamplified using the primers Tpconj-F1 and Tpconj-R1 and assembled into the p0521S-URA by yeast recombination as described above to give the final vector p0521-Tp-PEPCK-YFP.

### Sequence analysis of plasmids passaged through *P. tricornutum*

Plasmids isolated from *P. tricornutum* colonies transformed with plasmids containing large fragments of scaffold 25 (Supplementary Fig. 1) were transformed to *E. coli* and purified from agarose gels by RECO chips (Takara). Sequencing libraries were prepared for each plasmid for the Ion Torrent platform using the Ion Xpress Plus gDNA Fragment Library Kit (Life Technologies). Samples were barcoded, pooled and sequencing on an Ion Torrent 314 chip. Reads were mapped to the *P. tricornutum* genome using CLC Genomics Workbench and visualized using GenomeView^46^. Plasmid p0521s was sequenced after purification using the QIAprep kit using primers Seq0521smallF, Seq0521smallR, Seq0521smallF2, Seq0521smallF3, Seq0521smallF4 and Seq0521smallR2.

### Calculation of segregation efficiency for p0521s

Segregation efficiency (*S*_eff_) was calculated according to the following equation^47^:


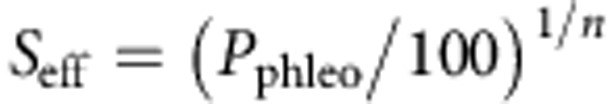


In this calculation, *P*_phleo_ is the percentage of phleomycin-resistant colonies in cultures passaged without selection and *n* is the number of nuclear division cycles estimated to have occurred during that time. To calculate *S*_eff_ for p0521s maintenance in *P. tricornutum*, a value of 30 was used for the variable *n* and the average *P*_phleo_ of 35 was used to arrive at an *S*_eff_ of 0.97.

### Quantitative PCR experiments

To create standard templates to calibrate the qPCR experiments, templates for each qPCR primer set were assembled onto plasmids. Plasmid pNorm1 was created by amplifying template regions for the yeast *HIS3* gene (primers pNorm1-1 and pNorm1-2), the chloramphenicol-resistance gene (CmR) from pCC1BAC (primers pNorm1-3 and pNorm1-4), *P. tricornutum* nitrate reductase (NR, Protein ID 54983, primers pNorm1-5 and pNorm1-6) and *P. tricornutum* urease gene (Ure, Protein ID 29702, primers pNorm1-7 and pNorm1-8), and assembling them into an EcoRI- and HindIII-digested pUC19 vector using Gibson assembly. Plasmid pNorm2 was created by first amplifying template regions for *P. tricornutum* RuBisCO small subunit located on the chloroplast chromosome (RbcS) (primers pNorm2-1 and pNorm2-2) and for *P. tricornutum* cytochrome B located on the mitochondrion chromosome (CytB) (primers pNorm2-3 and pNorm2-4). These amplified products were assembled into an EcoRI- and HindIII-digested pUC19 vector using Gibson assembly. Plasmids pNorm1 and pNorm2 were extracted from *E. coli* using Qiagen QIAprep kit and treated with Plasmid-Safe exonuclease (EpiCentre) to remove any residual genomic DNA. Treated plasmids were extracted twice with phenol:chloroform^48^, precipitated and quantified by Nanodrop (Thermo) to calibrate qPCR standard curves.

Quantitative PCR analysis was performed using 7900HT Fast Real-Time PCR System (Applied Biosystems) using Fast SYBR Green MasterMix (Applied Biosystems). Reactions consisted of master mix diluted to 1 × , plasmid or *P. tricornutum* genomic extract and 5 μM primers in 20 μl total volume. To perform qPCR experiments, total DNA was extracted from *P. tricornutum* cells containing plasmid p0521s or pPtPuc3 using a modified CTAB protocol^12^. Standard curves were performed for each primer pair using serial dilutions of pNorm1 or pNorm2 plasmid, as appropriate. The reactions were cycled under the following conditions: 95 °C for 20 s, 40 cycles of 95 °C for 1 s followed by 60 °C for 20 s during which data were collected. *C*t values were calculated by the SDS software, plotted as a function of number of template molecules and fit to a logarithmic trend line. Curves were linear over at least four orders of magnitude. Three biological replicates (plasmids extracted from different *P. tricornutum* strains containing plasmid) were tested, each with two to four technical replicates at each dilution. *C*t values resulting from experimental samples were used to calculate the number of molecules of template molecule in the qPCR reaction. The experiment was repeated at least twice for both p0521s and pPtPuc3 plasmids.

### Plasmid-safe experiments

Plasmids from *P. tricornutum* lines containing the p0521s plasmid were extracted using the modified alkaline lysis extraction protocol described above. A 2 × 2 factorial experimental design was set up to test the effects of restriction digest and exonuclease treatment on the ability of plasmids extracted from *P. tricornutum* to transform *E. coli*. First, ClaI restriction digest or mock reaction was performed on samples using 1–2 μg total DNA in a 100-μl total reaction with 1 × CutSmart buffer and ClaI (NEB) or water in digested or mock-digested samples, respectively. Reactions were incubated for 1 h at 37 °C. To each 100 μl digest or mock digest, 10 μl Plasmid-Safe reaction buffer was added and 8 μl ATP solution and 3 μl Plasmid-safe exonuclease or water (mock) to a final volume of 200 μl. The reactions were incubated at 37 °C for 1 h. Finally, the reactions were incubated at 70 °C for 1 h to inactivate the enzymes. Treated DNA was precipitated, resuspended in water and transformed into *E. coli* strain Epi300.

### Southern blot

For Southern blot analysis of *P. tricornutum* genomic DNA from lines containing the p0521s plasmid, DNA was extracted using a modified CTAB protocol^12^ from *P. tricornutum* cultures grown on L1 agar containing phleomycin (20 μg ml^−1^). DNA (∼30 μg) was digested with ClaI that cuts a single time within the p0521s plasmid and still cuts frequently within the *P. tricornutum* genome. Plasmid control DNA from *E. coli* was digested with RsrII that also cuts a single time within the p0521s sequence but is not affected by Dam methylation. Digested DNA was separated by agarose gel electrophoresis overnight at 0.1 V cm^−1^ in a 1% gel. Gels were treated for Southern blot by rinsing once in 0.25 M HCl, twice in denaturing solution (1.5 M NaCl, 0.5 M NaOH) and twice in neutralization solution (1.5 M NaCl, 0.5 M Tris, pH 7.0). Overnight transfers onto Hybond N+ membrane (GE) were performed in 20 × saline-sodium citrate (3 M NaCl, 300 mM Na_3_C_6_H_5_O_7_). Hybridization was performed using a probe to the ShBle cassette constructed by the DIG PCR Probe Synthesis Kit (Roche) according to the manufacturer's instruction using primers SB2 and 3′ShBle (Supplementary Table 1). Blots were developed using the bioluminescent substrate CDP-star and imaged on the C-DiGit chemiluminescent scanner (Licor).

### Laser scanning confocal microscopy

A Leica TCS SP5 confocal laser scanning microscope equipped with a × 100 oil immersion objective was used to visualize the fluorescently tagged proteins. CFP, GFP and YFP were excited with 458, 488 and 514 nm lasers, with emission monitored at 470–520 (CFP), 505–530 nm (GFP) and 525–560 nm (YFP). Autoflourescence of the plastid was monitored at 700–740 nm.

### SEM microscopy—sample preparation protocol

Cells were spun at room temperature for 4 min at 2,000*g* to produce a loose pellet. Media (950 μl) were removed and replaced with 1 ml of fixative. The fixative solution was 2.5% glutaraldehyde, 100 mM sodium cacodylate, 2 mM calcium chloride and 2% sucrose (fixative was added cold and samples were stored at 4 °C). Cells were immobilized on polyethylenimine or poly-D-lysine coated ITO glass coverslips for 2 min and washed in 0.1 M cacodylate buffer with 2 mM calcium chloride and 2% sucrose for 5 × 2 min on ice. Cells were post fixed in 2% osmium tetoxide with 2% sucrose in 0.1 M cacodylate for 30 min on ice. Cells were rinsed in double distilled water and dehydrated in an ethanol series (20, 50, 70, 100%) for 2 min each on ice. Samples were critical point dried (with CO_2_) and sputter-coated with a thin layer of Au/Pd. Samples were imaged with a Zeiss Merlin Fe-SEM at 2.5 kev, 83 pA probe current and 2.9 mm working distance (zero tilt) using the in-lens SE detector.

## Author contributions

B.J.K. discovered the first replicating plasmids in *P. tricornutum* (p0521 and p0521s), discovered conjugation and PEG-mediated transformation methods in *P. tricornutum*, optimized conjugation methods and tested plasmid components responsible for episome replication. R.E.D. developed optimized diatom conjugation methods and tested plasmid components responsible for episome replication. S.C.L. customized p0521s to confer nourseothricin resistance, express YFP-tagged protein in *T. pseudonana* and established conjugation in this diatom. A.P.R.P., C.M.N., J.K.B., M.H.E., H.O.S., C.A.H., J.I.G., J.C.V., A.E.A., C.L.D. and P.D.W. conceived and designed the additional experiments. J.M., A.P.R.P., C.M.N., J.K.B., T.J.D., J.J., K.B., J.T.F.G. and P.D.W. performed additional experiments. B.J.K., R.E.D., S.C.L., J.M., A.P.R.P., C.M.N., J.K.B., R.E.V., C.L.D. and P.D.W. analysed the data. B.J.K., S.C.L., A.E.A., C.L.D. and P.D.W. wrote the paper.

## Additional information

**How to cite this article:** Karas, B. J. *et al*. Designer diatom episomes delivered by bacterial conjugation. *Nat. Commun*. 6:6925 doi: 10.1038/ncomms7925 (2015).

## Supplementary Material

Supplementary Information Supplementary Figures 1-9, Supplementary Tables 1-4.
