# Supplementary material for: Designer diatom episomes delivered by bacterial conjugation
Source: Nat Commun. 2015 Apr 21;6:6925. doi: 10.1038/ncomms7925 (PMC4411287; doi:10.1038/ncomms7925)
Supplement: Supplementary Information — Supplementary Figures 1-9, Supplementary Tables 1-4. [file ncomms7925-s1.pdf]

Supplementary Figure 1

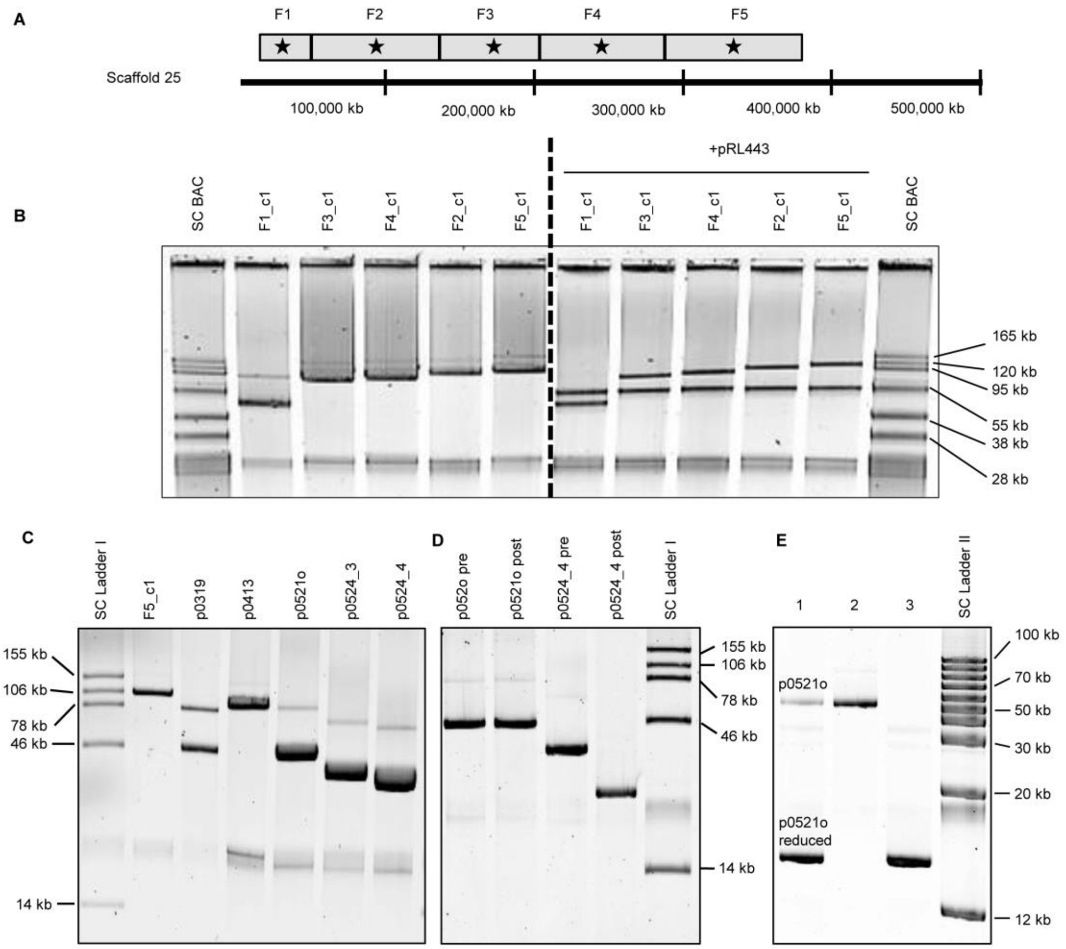

Cloning of five fragments of *P. tricornutum* scaffold 25.**A.** Map of fragments F1–F5 on scaffold 25. **B.** Each fragment (F1-F5) was cloned in yeast and moved to *E. coli* strain Epi300 with or without the conjugative plasmid pRL443. Plasmids were isolated from *E. coli* and separated by agarose gel electrophoresis to verify correct size. **C.** Episomes rescued in *E. coli* from *P. tricornutum* lines obtained from PEG-mediated and electroporation-based transformation were extracted and DNA was separated by agarose gel electrophoresis. **D.** Plasmids p0521o and p0524_4 were subjected to a second round of *P. tricornutum* transformation and episome rescue in *E. coli* for subsequent isolation. Plasmids before (pre) and after (post) the second passage through *P. tricornutum* were separated by agarose gel electrophoresis. **E.** Identification of minimized version of plasmid p0521o. After long-term stationary phase growth, DNA isolated from *P. tricornutum* was transformed into *E. coli* and plasmids from the pool of resulting colonies were extracted and separated by agarose gel electrophoresis (lane 1). Individual *E. coli* clones were identified that contained either the original p0521o plasmid (lane 2) or the minimized version, p0521o reduced (lane 3).

Supplementary Figure 2

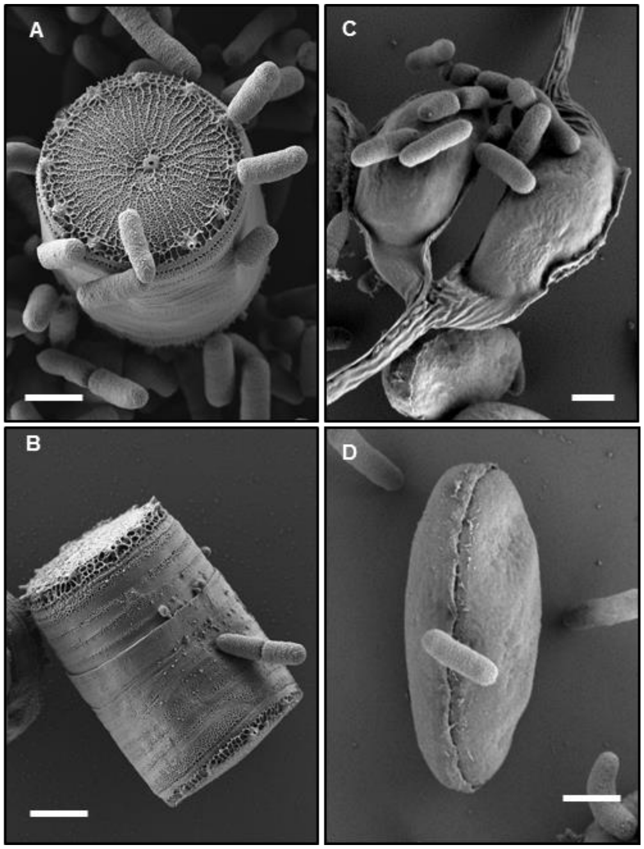

Diatoms form associations with *E. coli*. Scanning electron microscopic (SEM) images of *T. pseudonana* (A and B) and *P. tricornutum* (C and D) cells after the 90 minute conjugation incubation at 30° C with *E. coli* containing p0521s and pTA-MOB. Image C shows a dividing *P. tricornutum* cell that is in the process of changing to the ovoid morphotype. Scale bar indicates 1 μm.

Supplementary Figure 3

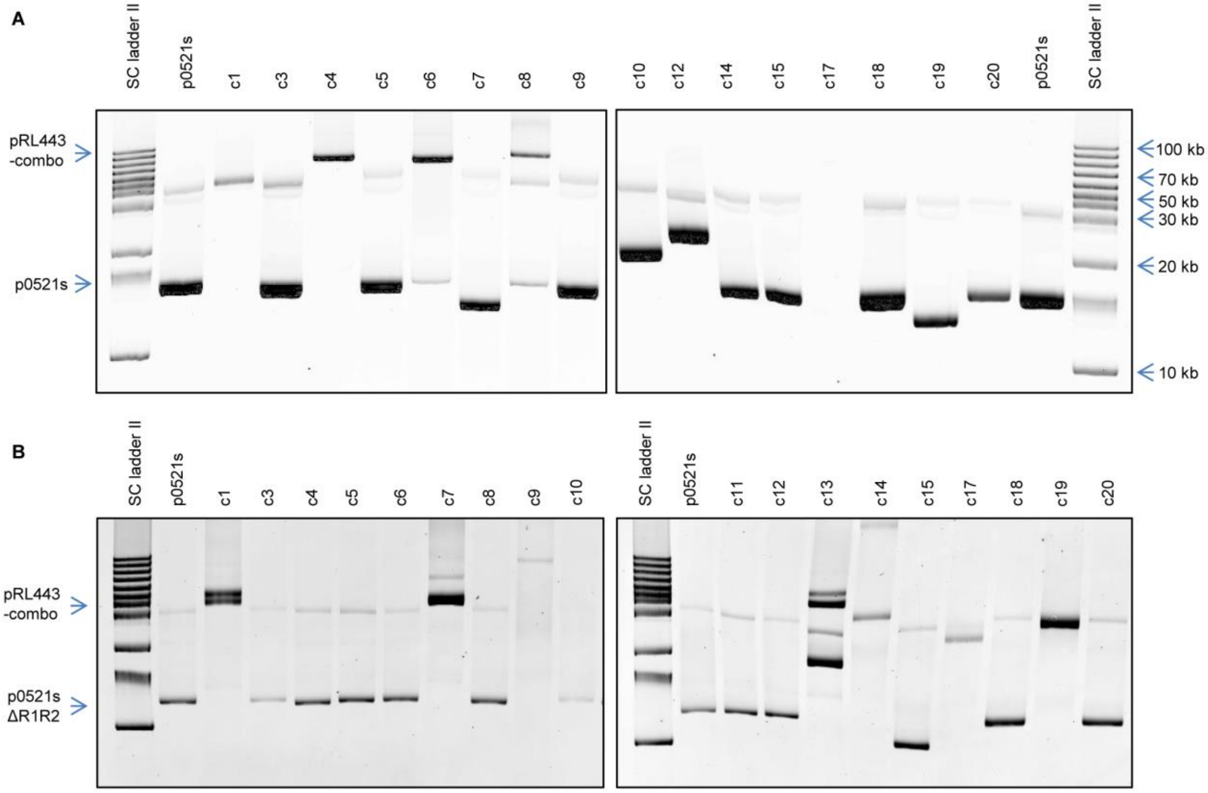

Episomes rescued from *P. tricornutum* exconjugant lines. **A.** DNA from 16 exconjugant *P. tricornutum* lines containing plasmid p0521s was transferred to *E. coli* and plasmids were isolated and separated by agarose gel electrophoresis. **B.** Plasmids were prepared as in part A but *P. tricornutum* was conjugated with plasmid p0521s-ΔR1R2.

Supplementary Figure 4

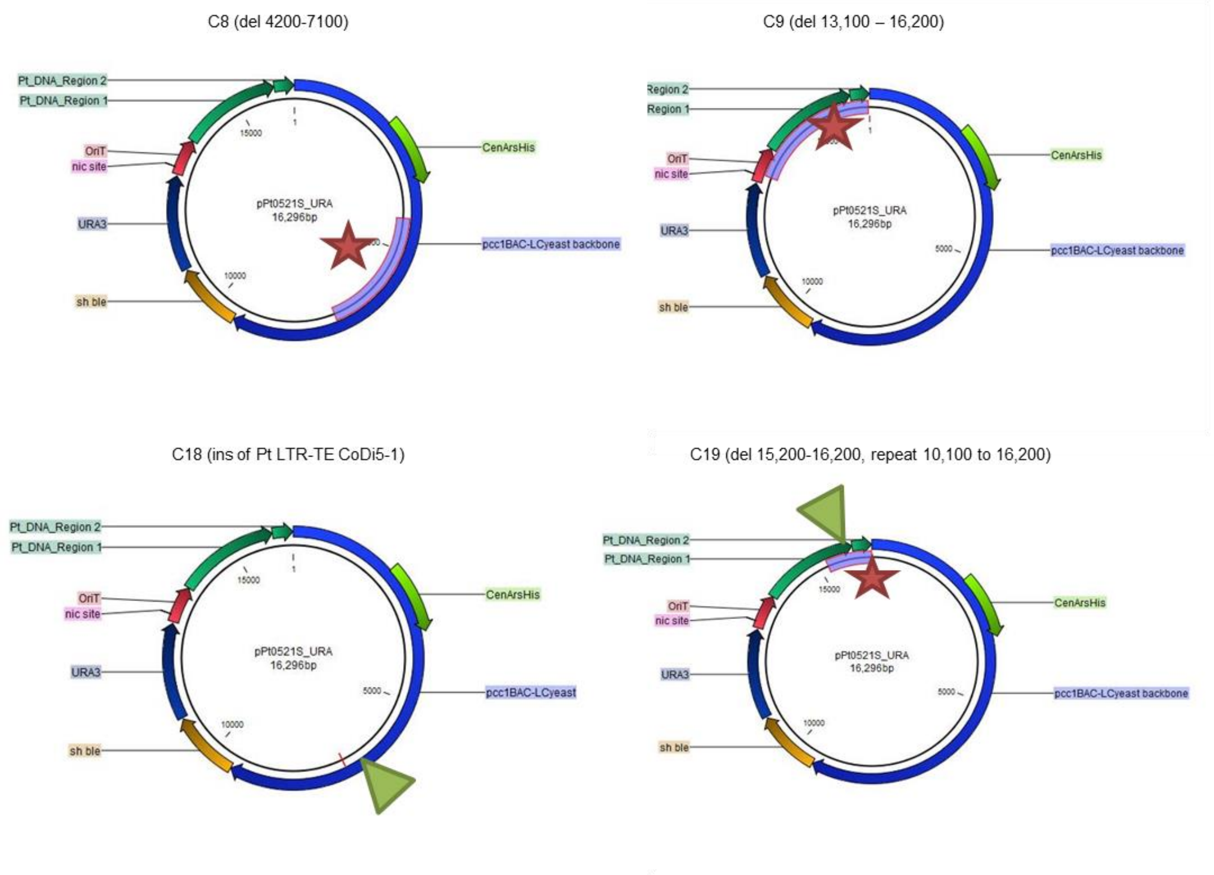

Results of sequencing p0521s plasmids rescued from *P. tricornutum* exconjugants. Various deletions (e.g. C8 and C9, red star with blue highlighted regions) and insertions (e.g. c18 and c19, green triangle) were detected by Sanger DNA sequencing.

Supplementary Figure 5

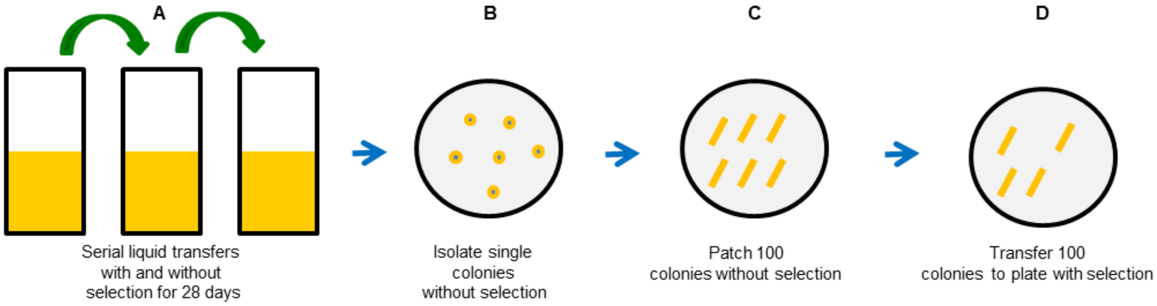

E

	Day 18		Day 28		Day 38	
Experiment	Plates - selection	Plates + selection	Plates - selection	Plates + selection	Plates - selection	Plates + selection
1. Passage without selection	30	21	30	12	30	14
2. Passaged with selection	30	30	30	30	30	30

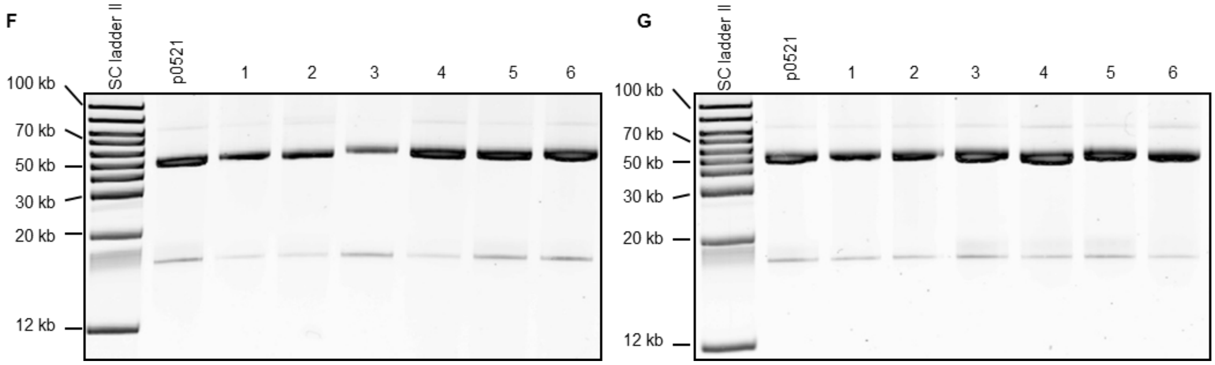

Outline of the experimental setup to test p0521s and p0521 maintenance in *P. tricornutum* lines grown in the absence of antibiotic selection. *P. tricornutum* lines containing p0521s were grown with or without selection for 28 days during which time they were serially diluted one hundred-fold at three different times (**A**). After 28 days, culture was plated to obtain single colonies on non-selective medium (**B**) and patched on non-selective medium (**C**)**.** Colonies were replica patched on selective (phleomycin 20 µg ml^{-1}) solid L1 medium (**D**). **E.** Stability of plasmid p0521 in *P. tricornutum* lines after growth in seawater medium with or without antibiotic selection. The experiment was performed as outlined in part A, but for a total of 18, 28, or 38 days. **F.** Episome rescue from single *P. tricornutum* p0521 lines sub-cultured without (lanes 1-3) or with (lanes 4-6) antibiotic selection was performed after 18 (F) or 38 (G) days. After sub-culture, *P. tricornutum* lines were selected on solid medium with antibiotic selection and episomes were rescued and separated by agarose gel electrophoresis.

Supplementary Figure 6

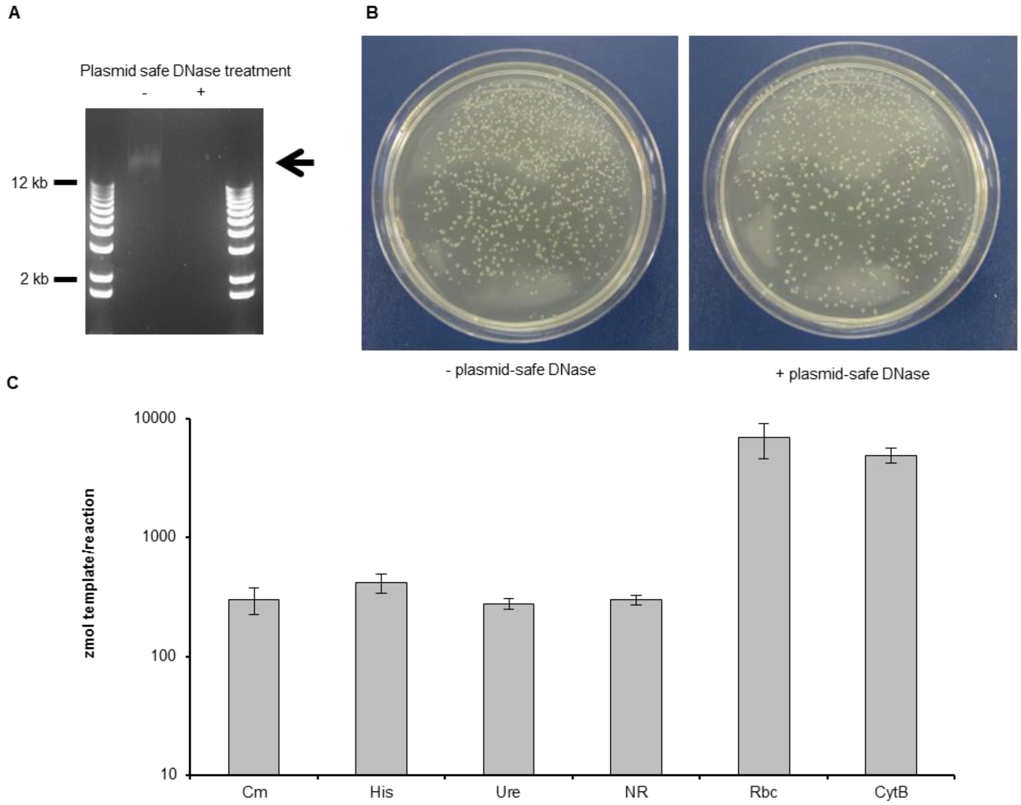

Plasmid p0521 replicates as a circle in *P. tricornutum* and with copy number equivalent to native chromosomes. **A.** DNA isolated from *P. tricornutum* lines containing p0521 was untreated or treated with Plasmid-safe exonuclease (Epicentre) and separated by agarose gel electrophoresis. Arrow indicates *P. tricornutum* genomic DNA that was degraded by the exonuclease treatment (+) but still present in control treatment (-). **B.** After transformation of the treated or control DNA from part A into *E. coli*, equivalent numbers of colonies were observed. **C.** Results of qPCR with DNA isolated from *P. tricornutum* lines containing p0521. See Fig. 2E for legend.

Supplementary Figure 7

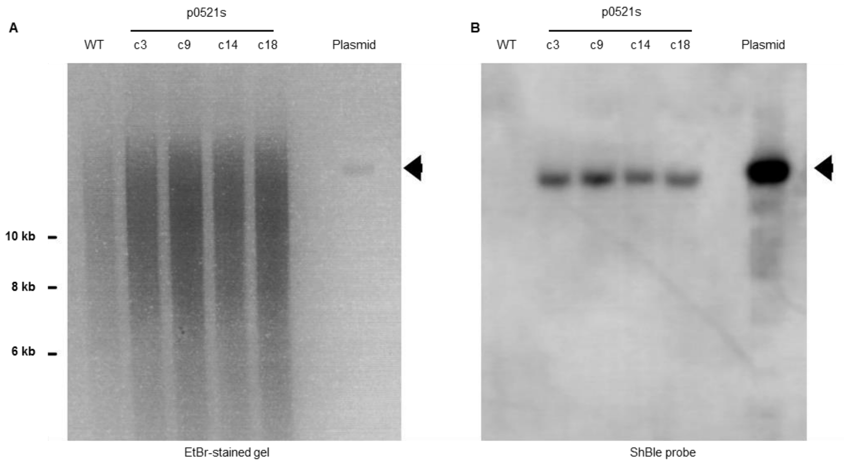

P. tricornutum genomic DNA was extracted from wild type (WT) or p0521s ex-conjugant lines (c3, c9, c14, and c18, see Extended Data Fig. 3A) and digested with ClaI that cuts a single time within p0521s. To avoid ClaI Dam methylation in *E. coli*-isolated plasmid controls, p0521s isolated from *E. coli* was digested with RsrII that cuts a single time within the plasmid. **A.** DNA (30 µg) was separated by agarose gel electrophoresis for 18 hr at 25 V to resolve higher molecular weight DNA and stained with ethidium bromide. The plasmid band is visible in the ethidium bromide-stained gel (arrow). **B.** Gels were blotted and hybridized with a probe to the ShBle gene. Only one band was observed on the ShBle-probed blot corresponding to the linearized plasmid position and consistent with lack of insertion of the plasmid elsewhere in the chromosome.

Supplementary Figure 8

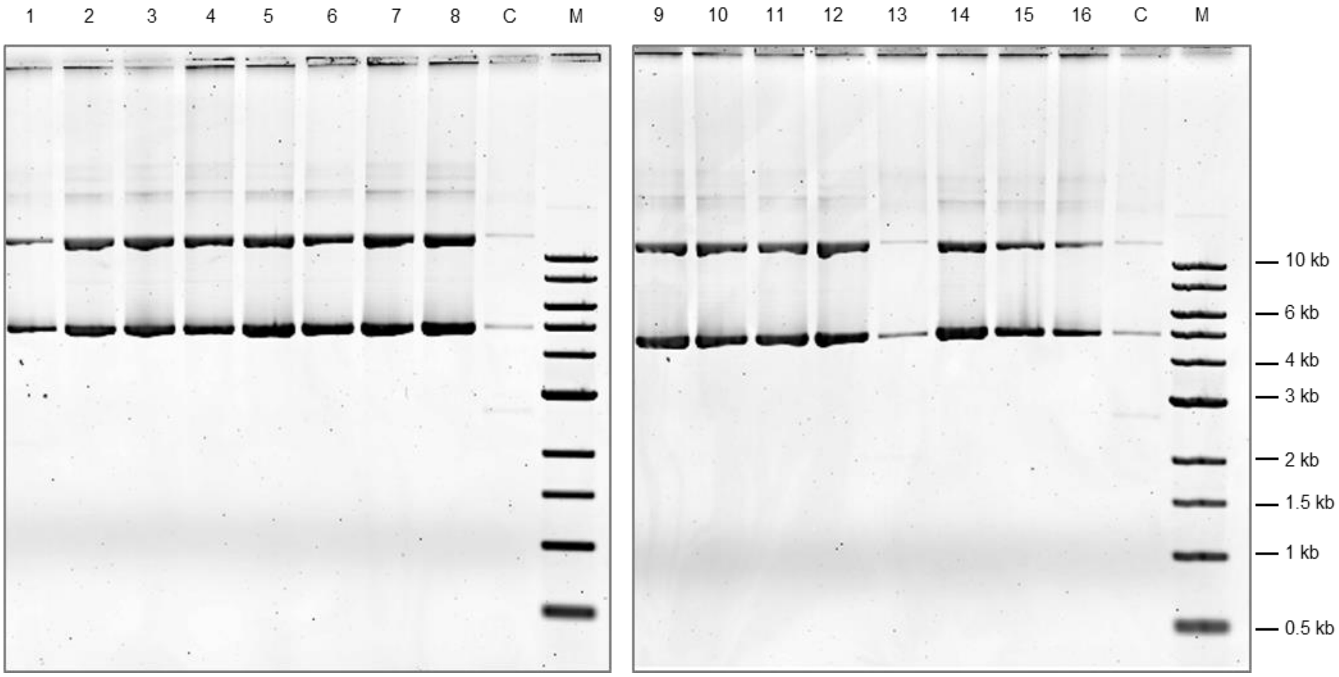

Plasmid pTpPuc3 rescued from *T. pseudonana* exconjugant lines. DNA from 16 *T. pseudonana* exconjugant lines was extracted and transformed to *E. coli* for subsequent isolation and separation by agarose gel electrophoresis. Lanes marked with "M" indicate 1-kb ladder (NEB) and with "C" indicate pTpPuc3 plasmid control.

Supplementary Figure 9

Sequence for pBK-RBYV. From 5' end: XhoI site (red), URA3 region (underline), XhoI site (red), pCCBac1-LCyeast (bold), ShBle marker (green: FcpF promoter bold, ShBle ORF underline, FcpA terminator bold), OriT (brown)

CTCGAGCGTTGCAGGCCATGCTGTCCAGGCAGGTAGATGACGACCATCAGGGACAGCTTCA
AGGATCGCTCGCGGCTCTTACCAGCCTAACTTCGATCACTGGACCGCTGATCGTCACGGCGA
TTTATGCCGCCTCGGCGAGCACATGGAACGGGTTGGCATGGATTGTAGGCGCCGCCCTATAC
CTTGTCTGCCTCCCCGCGTTGCGTCGCGGTGCATGGAGCCGGGCCACCTCGACCTGAATGGA
AGCCGGCGGCACCTCGCTAACGGATTCACCACTCCAAGAATTGGAGCCAATCAATTCTTGCG
GAGAACTGTGAATGCGCAAACCAACCCTTGGCAGAACATATCCATCGCGTCCGCCATCTCCA
GCAGCCGCACGCGGCGCATCCCCCCCCCCCTTTCAATTCAATTCATCATTTTTTTTTTTATTCTT
TTTTTTGATTTCGGTTTCTTTGAAATTTTTTTGATTCGGTAATCTCCGAACAGAAGGAAGAAC
GAAGGAAGGAGCACAGACTTAGATTGGTATATATACGCATATGTAGTGTTGAAGAAACATG
AAATTGCCCAGTATTCTTAACCCAACTGCACAGAACAAAAACCTGCAGGAAACGAAGATAA
ATCATGTCGAAAGCTACATATAAGGAACGTGCTGCTACTCATCCTAGTCCTGTTGCTGCCAA
GCTATTTAATATCATGCACGAAAAGCAAACAAACTTGTGTGCTTCATTGGATGTTCGTACCA
CCAAGGAATTACTGGAGTTAGTTGAAGCATTAGGTCCCAAAATTTGTTTACTAAAAACACAT
GTGGATATCTTGACTGATTTTTCCATGGAGGGCACAGTTAAGCCGCTAAAGGCATTATCCGC
CAAGTACAATTTTTTACTCTTCGAAGACAGAAAATTTGCTGACATTGGTAATACAGTCAAAT
TGCAGTACTCTGCGGGTGTATACAGAATAGCAGAATGGGCAGACATTACGAATGCACACGG
TGTGGTGGGCCCAGGTATTGTTAGCGGTTTGAAGCAGGCGGCAGAAGAAGTAACAAAGGAA
CCTAGAGGCCTTTTGATGTTAGCAGAATTGTCATGCAAGGGCTCCCTATCTACTGGAGAATA
TACTAAGGGTACTGTTGACATTGCGAAGAGCGACAAAGATTTTGTTATCGGCTTTATTGCTC
AAAGAGACATGGGTGGAAGAGATGAAGGTTACGATTGGTTGATTATGACACCCGGTGTGGG
TTTAGATGACAAGGGAGACGCATTGGGTCAACAGTATAGAACCGTGGATGATGTGGTCTCTA
CAGGATCTGACATTATTATTGTTGGAAGAGGACTATTTGCAAAGGGAAGGGATGCTAAGGT
AGAGGGTGAACGTTACAGAAAAGCAGGCTGGGAAGCATATTTGAGAAGATGCGGCCAGCA
AAACTAAAAAACTGTATTATAAGTAAATGCATGTATACTAAACTCACAAATTAGAGCTTCAA
TTTAATTATATCAGTTATTACTCGGGCGTAATGATTTTTATAATGACGAAAAAAAAAAAAATT
GGAAAGAAAAGGGGGGGGGGGCAGCGTTGGGTCCTGGCCACGGGTGCGCATGATCGTGCTC
CTGTCGTTGAGGACCCGGCTAGGCTGGCGGGGTTGCCTTACTGGTTAGCAGAATGAATCACC
GATACGCGAGCGAACGTGAAGCGACTGCTGCTGCAAAACGTCTGCGACCTGAGCAACAACA
TGAATGGTCTTCGGTTTCCGTGTTTCGTAAAGTCTGGAAACGCGGAAGTCAGCGCCCTGCAC
CATTATGTTCCGGATCTGCATCGCAGGATGCTGCTGGCTACCCTGTGGAACACCTACATCTGT
ATTAACGAAGCGCTGGCATTGACCCTGAGTGATTTTTCTCTGGTCCCGCCGCATCCATACCGC
CAGTTGTTTACCCTCACAACGTTCCAGTAACCGGGCATGTTCATCATCAGTAACCCGTATCGT
GAGCATCCTCTCTCGTTTCATCGCTCGAG**CTGGTTGCCCTCGCCGCTGGGCTGGCGGCCG**
TCTATGGCCCTGCAAACGCGCCAGAAACGCCGTCGAAGCCGTGTGCGAGACACCGCGG
CCGGCCGCCGGCGTTGTGGATACCTCGCGGAAAACTTGGCCCTCACTGACAGATGAGG
GGCGGACGTTGACACTTGAGGGGCCGACTCACCCGGCGCGGCGTTGACAGATGAGGG
GCAGGCTCGATTTCGGCCGGCGACGTGGAGCTGGCCAGCCTCGCAAATCGGCGAAAA
CGCCTGATTTTACGCGAGTTTCCCACAGATGATGTGGACAAGCCTGGGGATAAGTGCC
CTGCGGTATTGACACTTGAGGGGCGCGACTACTGACAGATGAGGGGCGCGATCCTTGA
CACTTGAGGGGCAGAGTGCTGACAGATGAGGGGCGCACCTATTGACATTTGAGGGGCT
GTCCACAGGCAGAAAATCCAGCATTTGCAAGGGTTTCCGCCCGTTTTTCGGCCACCGC
TAACCTGTCTTTTAACCTGCTTTTAAACCAATATTTATAAACCTTGTTTTTAACCAGGGC
TGCGCCCTGTGCGCGTGACCGCGCACGCCGAAGGGGGGTGCCCCCCCTTCTCGAACCC
TCCCGGTCGAGTGAGCGAGGAAGCACCAGGGAACAGCACTTATATATTCTGCTTACAC

**ACGATGCCTGAAAAAACTTCCCTTGGGGTTATCCACTTATCCACGGGGATATTTTTATA
ATTATTTTTTTTATAGTTTTTAGATCTTCTTTTTTAGAGCGCCTTGTAGGCCTTTATCCA
TGCTGGTTCTAGAGAAGGTGTTGTGACAAATTGCCCTTTCAGTGTGACAAATCACCCTC
AAATGACAGTCCTGTCTGTGACAAATTGCCCTTAACCCTGTGACAAATTGCCCTCAGAA
GAAGCTGTTTTTTCACAAAGTTATCCCTGCTTATTGACTCTTTTTTATTTAGTGTGACAA
TCTAAAAACTTGTCACACTTCACATGGATCTGTCATGGCGGAAACAGCGGTTATCAATC
ACAAGAAACGTAAAAATAGCCCGCGAATCGTCCAGTCAAACGACCTCACTGAGGCGGC
ATATAGTCTCTCCCGGGATCAAAAACGTATGCTGTATCTGTTCGTTGACCAGATCAGAA
AATCTGATGGCACCCTACAGGAACATGACGGTATCTGCGAGATCCATGTTGCTAAATAT
GCTGAAATATTCGGATTGACCTCTGCGGAAGCCAGTAAGGATATACGGCAGGCATTGA
AGAGTTTCGCGGGGAAGGAAGTGGTTTTTTATCGCCCTGAAGAGGATGCCGGCGATGA
AAAAGGCTATGAATCTTTTCCTTGGTTTATCAAACGTGCGCACAGTCCATCCAGAGGGC
TTTACAGTGTACATATCAACCCATATCTCATTCCCTTCTTTATCGGGTTACAGAACCGG
TTTACGCAGTTTCGGCTTAGTGAAACAAAAGAAATCACCAATCCGTATGCCATGCGTTT
ATACGAATCCCTGTGTCAGTATCGTAAGCCGGATGGCTCAGGCATCGTCTCTCTGAAA
ATCGACTGGATCATAGAGCGTTACCAGCTGCCTCAAAGTTACCAGCGTATGCCTGACTT
CCGCCGCCGCTTCCTGCAGGTCTGTGTTAATGAGATCAACAGCAGAACTCCAATGCGC
CTCTCATACATTGAGAAAAAGAAAGGCCGCCAGACGACTCATATCGTATTTTCCTTCCG
CGATATCACTTCCATGACGACAGGATAGTCTGAGGGTTATCTGTCACAGATTTGAGGG
TGGTTCGTCACATTTGTTCTGACCTACTGAGGGTAATTTGTCACAGTTTTGCTGTTTCC
TTCAGCCTGCATGGATTTTCTCATACTTTTTGAACTGTAATTTTTAAGGAAGCCAAATTT
GAGGGCAGTTTGTCACAGTTGATTTCCTTCTCTTTCCCTTCGTCATGTGACCTGATATC
GGGGGTTAGTTCGTCATCATTGATGAGGGTTGATTATCACAGTTTATTACTCTGAATTG
GCTATCCGCGTGTGTACCTCTACCTGGAGTTTTTCCCACGGTGGATATTTCTTCTTGCG
CTGAGCGTAAGAGCTATCTGACAGAACAGTTCTTCTTTGCTTCCTCGCCAGTTCGCTCG
CTATGCTCGGTTACACGGCTGCGGCGAGCATCACGTGCTATAAAAATAATTATAATTTA
AATTTTTTAATATAAATATATAAATTAAAAATAGAAAGTAAAAAAAGAAATTAAAGAAA
AAATAGTTTTTGTTTTCCGAAGATGTAAAAGACTCTAGGGGGATCGCCAACAAATACTA
CCTTTTATCTTGCTCTTCCTGCTCTCAGGTATTAATGCCGAATTGTTTCATCTTGTCTGT
GTAGAAGACCACACACGAAAATCCTGTGATTTTACATTTTACTTATCGTTAATCGAATG
TATATCTATTTAATCTGCTTTTCTTGTCTAATAAATATATATGTAAAGTACGCTTTTTGT
TGAAATTTTTTAAACCTTTGTTTATTTTTTTTTCTTCATTCCGTAACTCTTCTACCTTCTT
TATTTACTTTCTAAAATCCAAATACAAAACATAAAAATAAATAAACACAGAGTAAATTC
CCAAATTATTCCATCATTAAAAGATACGAGGCGCGTGTAAGTTACAGGCAAGCGATCCT
AGTACACTCTATATTTTTTTATGCCTCGGTAATGATTTTCATTTTTTTTTTTCCACCTAG
CGGATGACTCTTTTTTTTTCTTAGCGATTGGCATTATCACATAATGAATTATACATTATA
TAAAGTAATGTGATTTCTTCGAAGAATATACTAAAAAATGAGCAGGCAAGATAAACGAA
GGCAAAGATGACAGAGCAGAAAGCCCTAGTAAAGCGTATTACAAATGAAACCAAGATT
CAGATTGCGATCTCTTTAAAGGGTGGTCCCCTAGCGATAGAGCACTCGATCTTCCCAG
AAAAAGAGGCAGAAGCAGTAGCAGAACAGGCCACACAATCGCAAGTGATTAACGTCCA
CACAGGTATAGGGTTTCTGGACCATATGATACATGCTCTGGCCAAGCATTCCGGCTGG
TCGCTAATCGTTGAGTGCATTGGTGACTTACACATAGACGACCATCACACCACTGAAGA
CTGCGGGATTGCTCTCGGTCAAGCTTTTAAAGAGGCCCTACTGGCGCGTGGAGTAAAA
AGGTTTGGATCAGGATTTGCGCCTTTGGATGAGGCACTTTCCAGAGCGGTGGTAGATC
TTTCGAACAGGCCGTACGCAGTTGTCGAACTTGGTTTGCAAAGGGAGAAAGTAGGAGA
TCTCTCTTGCGAGATGATCCCGCATTTTCTTGAAAGCTTTGCAGAGGCTAGCAGAATTA
CCCTCCACGTTGATTGTCTGCGAGGCAAGAATGATCATCACCGTAGTGAGAGTGCGTT
CAAGGCTCTTGCGGTTGCCATAAGAGAAGCCACCTCGCCCAATGGTACCAACGATGTT
CCCTCCACCAAAGGTGTTCTTATGTAGTTTTACACAGGAGTCTGGACTTGACGCTAGTG
ATAATAAGTGACTGAGGTATGTGCTCTTCTTATCTCCTTTTGTAGTGTTGCTCTTATTTT
AAACAACTTTGCGGTTTTTTGATGACTTTGCGATTTTGTTGTTGCTTTGCAGTAAATTG**

**CAAGATTTAATAAAAAAACGCAAAGCAATGATTAAAGGATGTTCAGAATGAAACTCATG
GAAACACTTAACCAGTGCATAAACGCTGGTCATGAAATGACGAAGGCTATCGCCATTG
CACAGTTTAATGATGACAGCCCGGAAGCGAGGAAAATAACCCGGCGCTGGAGAATAGG
TGAAGCAGCGGATTTAGTTGGGGTTTCTTCTCAGGCTATCAGAGATGCCGAGAAAGCA
GGGCGACTACCGCACCCGGATATGGAAATTCGAGGACGGGTTGAGCAACGTGTTGGTT
ATACAATTGAACAAATTAATCATATGCGTGATGTGTTTGGTACGCGATTGCGACGTGCT
GAAGACGTATTTCCACCGGTGATCGGGGTTGCTGCCCATAAAGGTGGCGTTTACAAAA
CCTCAGTTTCTGTTCATCTTGCTCAGGATCTGGCTCTGAAGGGGCTACGTGTTTTGCTC
GTGGAAGGTAACGACCCCCAGGGAACAGCCTCAATGTATCACGGATGGGTACCAGATC
TTCATATTCATGCAGAAGACACTCTCCTGCCTTTCTATCTTGGGGAAAAGGACGATGTC
ACTTATGCAATAAAGCCCACTTGCTGGCCGGGGCTTGACATTATTCCTTCCTGTCTGGC
TCTGCACCGTATTGAAACTGAGTTAATGGGCAAATTTGATGAAGGTAAACTGCCCACC
GATCCACACCTGATGCTCCGACTGGCCATTGAAACTGTTGCTCATGACTATGATGTCAT
AGTTATTGACAGCGCGCCTAACCTGGGTATCGGCACGATTAATGTCGTATGTGCTGCT
GATGTGCTGATTGTTCCCACGCCTGCTGAGTTGTTTGACTACACCTCCGCACTGCAGTT
TTTCGATATGCTTCGTGATCTGCTCAAGAACGTTGATCTTAAAGGGTTCGAGCCTGATG
TACGTATTTTGCTTACCAAATACAGCAATAGCAATGGCTCTCAGTCCCCGTGGATGGAG
GAGCAAATTCGGGATGCCTGGGGAAGCATGGTTCTAAAAAATGTTGTACGTGAAACGG
ATGAAGTTGGTAAAGGTCAGATCCGGATGAGAACTGTTTTTGAACAGGCCATTGATCA
ACGCTCTTCAACTGGTGCCTGGAGAAATGCTCTTTCTATTTGGGAACCTGTCTGCAATG
AAATTTTCGATCGTCTGATTAAACCACGCTGGGAGATTAGATAATGAAGCGTGCGCCT
GTTATTCCAAAACATACGCTCAATACTCAACCGGTTGAAGATACTTCGTTATCGACACC
AGCTGCCCCGATGGTGGATTCGTTAATTGCGCGCGTAGGAGTAATGGCTCGCGGTAAT
GCCATTACTTTGCCTGTATGTGGTCGGGATGTGAAGTTTACTCTTGAAGTGCTCCGGG
GTGATAGTGTTGAGAAGACCTCTCGGGTATGGTCAGGTAATGAACGTGACCAGGAGCT
GCTTACTGAGGACGCACTGGATGATCTCATCCCTTCTTTTCTACTGACTGGTCAACAGA
CACCGGCGTTCGGTCGAAGAGTATCTGGTGTCATAGAAATTGCCGATGGGAGTCGCCG
TCGTAAAGCTGCTGCACTTACCGAAAGTGATTATCGTGTTCTGGTTGGCGAGCTGGAT
GATGAGCAGATGGCTGCATTATCCAGATTGGGTAACGATTATCGCCCAACAAGTGCTT
ATGAACGTGGTCAGCGTTATGCAAGCCGATTGCAGAATGAATTTGCTGGAAATATTTCT
GCGCTGGCTGATGCGGAAAATATTTCACGTAAGATTATTACCCGCTGTATCAACACCGC
CAAATTGCCTAAATCAGTTGTTGCTCTTTTTTCTCACCCCGGTGAACTATCTGCCCGGT
CAGGTGATGCACTTCAAAAAGCCTTTACAGATAAAGAGGAATTACTTAAGCAGCAGGC
ATCTAACCTTCATGAGCAGAAAAAAGCTGGGGTGATATTTGAAGCTGAAGAAGTTATC
ACTCTTTTAACTTCTGTGCTTAAAACGTCATCTGCATCAAGAACTAGTTTAAGCTCACG
ACATCAGTTTGCTCCTGGAGCGACAGTATTGTATAAGGGCGATAAAATGGTGCTTAAC
CTGGACAGGTCTCGTGTTCCAACTGAGTGTATAGAGAAAATTGAGGCCATTCTTAAGG
AACTTGAAAAGCCAGCACCCTGATGCGACCACGTTTTAGTCTACGTTTATCTGTCTTTA
CTTAATGTCCTTTGTTACAGGCCAGAAAGCATAACTGGCCTGAATATTCTCTCTGGGCC
CACTGTTCCACTTGTATCGTCGGTCTGATAATCAGACTGGGACCACGGTCCCACTCGTA
TCGTCGGTCTGATTATTAGTCTGGGACCACGGTCCCACTCGTATCGTCGGTCTGATTAT
TAGTCTGGGACCACGGTCCCACTCGTATCGTCGGTCTGATAATCAGACTGGGACCACG
GTCCCACTCGTATCGTCGGTCTGATTATTAGTCTGGGACCATGGTCCCACTCGTATCGT
CGGTCTGATTATTAGTCTGGGACCACGGTCCCACTCGTATCGTCGGTCTGATTATTAGT
CTGGAACCACGGTCCCACTCGTATCGTCGGTCTGATTATTAGTCTGGGACCACGGTCC
CACTCGTATCGTCGGTCTGATTATTAGTCTGGGACCACGATCCCACTCGTGTTGTCGGT
CTGATTATCGGTCTGGGACCACGGTCCCACTTGTATTGTCGATCAGACTATCAGCGTGA
GACTACGATTCCATCAATGCCTGTCAAGGGCAAGTATTGACATGTCGTCGTAACCTGTA
GAACGGAGTAACCTCGGTGTGCGGTTGTATGCCTGCTGTGGATTGCTGCTGTGTCCTG
CTTATCCACAACATTTTGCGCACGGTTATGTGGACAAAATACCTGGTTACCCAGGCCGT
GCCGGCACGTTAACCGGGCTGCATCCGATGCAAGTGTGTCGCTGTCGACGAGCTCGCG**

AGCTCGGACATGAGGTTGCCCCGTATTCAGTGTCGCTGATTTGTATTGTCTGAAGTTGT
TTTTACGTTAAGTTGATGCAGATCAATTAATACGATACCTGCGTCATAATTGATTATTT
GACGTGGTTTGATGGCCTCCACGCACGTTGTGATATGTAGATGATAATCATTATCACTT
TACGGGTCCTTTCCGGTGATCCGACAGGTTACGGGGCGGCGACCTCGCGGGTTTTCGC
TATTTATGAAAATTTTCCGGTTTAAGGCGTTTCCGTTCTTCTTCGTCATAACTTAATGTT
TTTATTTAAAATACCCTCTGAAAAGAAAGGAAACGACAGGTGCTGAAAGCGAGCTTTTT
GGCCTCTGTCGTTTCCTTTCTCTGTTTTTGTCCGTGGAATGAACAATGGAAGTCCGAGC
TCATCGCTAATAACTTCGTATAGCATACATTATACGAAGTTATATTCGATGCGGCCGCA
AGGGGTTCGCGTCAGCGGGTGTTGGCGGGTGTCGGGGCTGGCTTAACTATGCGGCAT
CAGAGCAGATTGTACTGAGAGTGCACCATATGCGGTGTGAAATACCACACAGATGCGT
AAGGAGAAAATACCGCATCAGGCGCCATTCGCCATTCAGCTGCGCAACTGTTGGGAAG
GGCGATCGGTGCGGGCCTCTTCGCTATTACGCCAGCTGGCGAAAGGGGGATGTGCTGC
AAGGCGATTAAGTTGGGTAACGCCAGGGTTTTCCCAGTCACGACGTTGTAAAACGACG
GCCAGTGAATTGTAATACGACTCACTATAGGGCGAATTCGAGCTCGGTACCCGGGGAT
CCTCTAGAGTCGACCTGCAGGCATGCAAGCTTGAGTATTCTATAGTCTCACCTAAATAG
CTTGGCGTAATCATGGTCATAGCTGTTTCCTGTGTGAAATTGTTATCCGCTCACAATTC
CACACAACATACGAGCCGGAAGCATAAAGTGTAAAGCCTGGGGTGCCTAATGAGTGAG
CTAACTCACATTAATTGCGTTGCGCTCACTGCCCGCTTTCCAGTCGGGAAACCTGTCGT
GCCAGCTGCATTAATGAATCGGCCAACGCGAACCCCTTGCGGCCGCCCGGGCCGTCGA
CCAATTCTCATGTTTGACAGCTTATCATCGAATTTCTGCCATTCATCCGCTTATTATCAC
TTATTCAGGCGTAGCAACCAGGCGTTTAAGGGCACCAATAACTGCCTTAAAAAAATTAC
GCCCCGCCCTGCCACTCATCGCAGTACTGTTGTAATTCATTAAGCATTCTGCCGACATG
GAAGCCATCACAAACGGCATGATGAACCTGAATCGCCAGCGGCATCAGCACCTTGTCG
CCTTGCGTATAATATTTGCCCATGGTGAAAACGGGGGCGAAGAAGTTGTCCATATTGG
CCACGTTTAAATCAAAACTGGTGAAACTCACCCAGGGATTGGCTGAGACGAAAAACAT
ATTCTCAATAAACCCTTTAGGGAAATAGGCCAGGTTTTCACCGTAACACGCCACATCTT
GCGAATATATGTGTAGAAACTGCCGGAAATCGTCGTGGTATTCACTCCAGAGCGATGA
AAACGTTTCAGTTTGCTCATGGAAAACGGTGTAACAAGGGTGAACACTATCCCATATCA
CCAGCTCACCGTCTTTCATTGCCATACGAAATTCCGGATGAGCATTCATCAGGCGGGC
AAGAATGTGAATAAAGGCCGGATAAAACTTGTGCTTATTTTTCTTTACGGTCTTTAAAA
AGGCCGTAATATCCAGCTGAACGGTCTGGTTATAGGTACATTGAGCAACTGACTGAAA
TGCCTCAAAATGTTCTTTACGATGCCATTGGGATATATCAACGGTGGTATATCCAGTGA
TTTTTTTCTCCATTTTAGCTTCCTTAGCTCCTGAAAATCTCGATAACTCAAAAAATACGC
CCGGTAGTGATCTTATTTCATTATGGTGAAAGTTGGAACCTCTTACGTGCCGATCAACG
TCTCATTTTCGCCAAAAGTTGGCCCAGGGCTTCCCGGTATCAACAGGGACACCAGGAT
TTATTTATTCTGCGAAGTGATCTTCCGTCACAGGTATTTATTCGCGATAAGCTCATGGA
GCGGCGTAACCGTCGCACAGGAAGGACAGAGAAAGCGCGGATCTGGGAAGTGACGGA
CAGAACGGTCAGGACCTGGATTGGGGAGGCGGTTGCCGCCGCTGCTGCTGACGGTGT
GACGTTCTCTGTTCCGGTCACACCACATACGTTCCGCCATTCCTATGCGATGCACATGC
TGTATGCCGGTATACCGCTGAAAGTTCTGCAAAGCCTGATGGGACATAAGTCCATCAG
TTCAACGGAAGTCTACACGAAGGTTTTTGCGCTGGATGTGGCTGCCCGGCACCGGGTG
CAGTTTGCGATGCCGGAGTCTGATGCGGTTGCGATGCTGAAACAATTATCCTGAGAAT
AAATGCCTTGGCCTTTATATGGAAATGTGGAACTGAGTGGATATGCTGTTTTTGTCTGT
TAAACAGAGAAGCTGGCTGTTATCCACTGAGAAGCGAACGAAACAGTCGGGAAAATCT
CCCATTATCGTAGAGATCCGCATTATTAATCTCAGGAGCCTGTGTAGCGTTTATAGGAA
GTAGTGTTCTGTCATGATGCCTGCAAGCGGTAACGAAAACGATTTGAATATGCCTTCAG
GAACAATAGAAATCTTCGTGCGGTGTTACGTTGAAGTGGAGCGGATTATGTCAGCAAT
GGACAGAACAACCTAATGAACACAGAACCATGATGTGGTCTGTCCTTTTACAGCCAGT
AGTGCTCGCCGCAGTCGAGCGACAGGGCGAAGCCCACCATGATTACGCCAAGCTCGAAA
TTAACCCTCACTAAAGGAACAAAAGCTGGTACCTAA**CAGGATTAGTGCAATTCGAGTTG**
AATCACTGGGAAAAACATTGTCTTCTTTTTTATATTATCATTTGCATTAGTGCTGCAGTC

GTAGATACTTGTTGGTTGAAAGACATCAGCTGGGAGGGACTGGACTAGCGTTTGGTAA
GGAGACATACCTGTTAACGTTGGTTGCAAAATTCCATTTCGCGATTTATGTTATCTGTA
AATCCTGATTTGTCTGGAATTCTTGATACTTCCGTTTTTTTAGAGGCCAATGATTAGCA
TCGGCGATTCTCAAAATAGCATTTTCGACATGCGGTGCTGATTTCATAAACATAGACAA
CGCTTTTACATGTAAAAGTAACTTGCGGACTTGGAACAGTGCTCTGTTTTTGGTGTGAA
CGTAACTCAGCAATATTTCTGTGCTAGCAAGGTTTTTTATGATCGACCGAAGATCTCAA
AACTCCGGGTCTTTCAACTGTCTGACTAGACCATGTTCGTAACGTCGGACAGCAGCTTT
CGTTGTACTGGTAGAATTTCTACGTGCGAAGCACGTGTAGGCAGGTTGAACGACGATC
CCTGCCGATGGATGGATTGGCACGCGGCGGAACGCTTTCGTGATCTACACCACCTGGA
TCTTCACATATCTTCGAAATCGAAAAATTAACCAAGTCGACGGTATCGATAATATTCTAGC
TGAGGGTACCCATGGCCAAGTTGACCAGTGCCGTTCCGGTGCTCACCGCGCGCGACGTCGCC
GGAGCGGTCGAGTTCTGGACCGACCGGCTCGGGTTCTCCCGGGACTTCGTGGAGGACGACTT
CGCCGGTGTGGTCCGGGACGACGTGACCCTGTTCATCAGCGCGGTCCAGGACCAGGTGGTGC
CGGACAACACCCTGGCCTGGGTGTGGGTGCGCGGCCTGGACGAGCTGTACGCCGAGTGGTC
GGAGGTCGTGTCCACGAACTTCCGGGACGCCTCCGGGCCGGCCATGACCGAGATCGGCGAG
CAGCCGTGGGGGCGGGAGTTCGCCCTGCGCGACCCGGCCGGCAACTGCGTGCACTTCGTGG
CCGAGGAGCAGGACTGACCGACGCCGACCAACACCGCCGGTCCGACGCGGCCCGACGGGTC
CGAGGCCTCGGAGATCTGGGCCCATGCGG**CCGCAACAACTACCTCGACTTTGGCTGGGAC**
ACTTTCAGTGAGGACAAGAAGCTTCAGAAGCGTGCTATCGAACTCAACCAGGGACGTG
CGGCACAAATGGGCATCCTTGCTCTCATGGTGCACGAACAGTTGGGAGTCTCTATCCT
TCCTTAAAAATTTAATTTTCATTAGTTGCAGTCACTCCGCTTTGGTTTCACAGTCAGGAA
TAACACTAGCTCGTCTTCACATCTTCCGCTGCATAACCCTGCTTCGGGGTCATTATAGCGATT
TTTTCGGTATATCCATCCTTTTTCGCACGATATACAGGATTTTGCCAAAGGGTTCGCGTAGAC
TTTCCTTGGTGTATCCAACGGCGTCAGCCGGGCAGGATAGGTGAAGTAGGCCCACCCGCGAG
CGGGTGTTCCTTCTTCACTGTCCCTTATTCGCACCTGGCGGTGCTCAACGGGAATCCTGCTCT
GCGAGGCTGGCCGGCTACCGCCGGCGTAACAGATGAGGGCAAGCGGATGGCTGATGAAACC
AAGCCAACCAGGAAGGGCAGCCCACCTATCAAGGTGTACTGCCTTCCAGACGAACGAAGAG
CGATTGAGGAAAAGGCGGCGGCGGCCGGCATGAGCCTGTCGGCCTACCTGCTGGCCGTCGG
CCAGGGCTACAAAATCACGGGCGTCGTGGACTATGAGCACGTCCGCGAGCTGGCCCGCATC
AATGGCGACCTGGGCCGCCTGGGCGGCCTGCTGAAACTCTGGCTCACCGACGACCCGCGCA
CGGCGCGGTTCGGTGATGCCACGATCCTCGCCCTGCTGGCGAAGATCGAAGAGAAGCAGGA
CGAGCTTGGCAAGGTCATGATGGGCGTGGTCCGCCCGAGGGCAGAGCCATGACTTTTTCAC
GTAACGGATCGGGGTGCGCGTGATTCGGAAGCACGTTCCATGGCCTCCATCAAGAAGAGGC
ACTTCGAGCTGTAAGTACATCACCGACGAGCAAGGCAAGACGATC

Supplemental Table 1: Oligonucleotides used in this study

PUC-ORIT-1	ACTATGCGGCATCAGAGCAGATTGTACTGAGAGTGCACCAGATCGTCTTGCCTTGCTCGT
PUC-ORIT-2	CGCCCTTCCCAACAGTTGCGCAGCCTGAATGGCGAATGGCATCTTCCGCTGCATAACCCT
PUC-ORIT-KM1	CATGCCATCCGTAAGATGCTTTTCTGTGACTGGTGAGTCGATTTATTCAACAAAGCCACG
PUC-ORIT-KM2	CGCCGCATACACTATTCTCAGAATGACTTGGTTGAGTGCCAGTGTTACAACCAATTAACC
PTPUC-1A	GGGTTTTCCCAGTCACGACGTTGTAAAACGACGGCCAGTGACCATGATTACGCCAAGCTC
PTPUC-2B	TTTGGTTTCACAGTCAGGAATAACACTAGCTCGTCTTCACGCGCCTGTTTTATTGAGAAC
PTPUC-3C	GCATCAACAGCCACGGGCCACCATCGACGAATAGACTCGGGCGAGCATCACGTGCTATAA
PTPUC-5D	GATTACGCCAAGCTTGCATGCCTGCAGGTCGACTCTAGAGCCGAGTCTATTCGTCGATGG
PTPUC-6C	CTTTGGTTTCACAGTCAGGAATAACACTAGCTCGTCTTCAGCGAGCATCACGTGCTATAA
PTPUC-7D	CGCCAAGCTTGCATGCCTGCAGGTCGACTCTAGAGTGAAGACGAGCTAGTGTTATTCCTG
PTPUC-8A	CCAGTCACGACGTTGTAAAACGACGGCCAGTGGATCCCTTTGTATTCAAAATAAGTATCG
PTPUC-12E	CCAACACGAACAACGTTCTCAATAAAACAGGCGCGTGAAGACGAGCTAGTGTTATTCCTG
PTPUC-13F	AATTTAAATTATAATTATTTTTATAGCACGTGATGCTCGCCCGAGTCTATTCGTCGATGG
PTPUC-14D	ACGCCAAGCTTGCATGCCTGCAGGTCGACTCTAGAGGTCAAGTCCAGACTCCTGTGTAAA
PTPUC-15F	AAATTATAATTATTTTTATAGCACGTGATGCTCGCTGAAGACGAGCTAGTGTTATTCCTG
PTPUC-17F	TTAAATTATAATTATTTTTATAGCACGTGATGCTCGCCTGGAAGCATGAAGTACTGCCAT
HA-MTUT-YFP-TERM-4	GCACCACCCCGGTGAACAGCTCCTCGCCCTTGCTCACCATCTGGTTTTGTTCCGGCGAAT
HA-MTUT-YFP-TERM-5	CTTGAACTAGCGACGAATCCGCATTCGCCGGAACAAAACCAGATGGTGAGCAAGGGCGAG
HA-MTUT-YFP-TERM-6	GTCCTCACTGAAAGTGTCCCAGCCAAAGTCGAGGTAGTTACTTGTACAGCTCGTCCATGC
HA-MTUT-YFP-TERM-7	CGCCGGGATCACTCTCGGCATGGACGAGCTGTACAAGTAACTACCTCGACTTTGGCTGGG
HA-MTUT-YFP-TERM-8	CACACAGGAAACAGCTATGACCATGATTACGCCAGTTGAAGACGAGCTAGTGTTATTCCT
NEWXFP-1	GGGTTTTCCCAGTCACGACGTTGTAAAACGACGGCCAGTGAGCGAACAGAAGACCCAATC
NEWXFP-2	GCAAGGCCAGTGCTGCTACGGCAATTCTCATGAATATCATCGTTCGCACAAGTGGTGACT
NEWXFP-9	TTCCCAGTCACGACGTTGTAAAACGACGGCCAGTGAATCTCGCCTATTCATGGTGTATAC
NEWXFP-10	TCCCAAAGATACGTCTACCGTAGCGACCGTCGAGGCTGCGCATAAGGGGGCGGCCGCGGA
NEWXFP-11	TCACAAGTTTGTACAAAAAAGCAGGCTCCGCGGCCGCCCCCTTATGCGCAGCCTCGACGG
NEWXFP-12	CACCACCCCGGTGAACAGCTCCTCGCCCTTGCTCACCATCGTTCGCACAAGTGGTGACTT
NEWXFP-13	TCCAGCAACCATTCTCATTCAAAGTCACCACTTGTGCGAACGATGGTGAGCAAGGGCGAG
NEWXFP-14	AAAGTGCAAAATTGATATGACAATCAGTCAAGAACTTTTACTTGTACAGCTCGTCCATGC
NEWXFP-15	TCACTCTCGGCATGGACGAGCTGTACAAGTAAAAGTTCTTGACTGATTGTCATATCAATT
NEWXFP-16	TTTCACACAGGAAACAGCTATGACCATGATTACGCCACAGACTGCTTTAGAGGAAGACGG
YEASTXFP-F2	ATAACACTAGCTCGTCTTCACGTAACTATAACGGTCCTAAAGGGCGAATTGGAGCTCC
YEASTXFP-F4	ATAACACTAGCTCGTCTTCACGTAACTATAACGGTCCTAAAGCGAACAGAAGACCCAATC
YEASTXFP-F5	ATAACACTAGCTCGTCTTCACGTAACTATAACGGTCCTAAAATCTCGCCTATTCATGGTGTATAC
YEASTXFP-R1	GTTATGCAGCGGAAGATCTATATTACCCTGTTATGTTGAAGACGAGCTAGTGTTATTCCT
YEASTXFP-R2	AGGGTTATGCAGCGGAAGATCTATATTACCCTGTTATCAGACTGCTTTAGAGGAAGACGG
SEQ0521SMALLF	GAAGAGGCACTTCGAGCTGT
SEQ0521SMALLR	TCAGTGAGGGCCAAGTTTTC
SEQ0521SMALLF2	GCATGCCAGGTAAGTCATGGAT
SEQ0521SMALLF3	GAACACAATTGTGGCAATGTTC
SEQ0521SMALLF4	CATCAAGGGACGTGCCAATCCA
SEQ0521SMALLR2	GCAAGTGCGAAGATCAGTTCAG
QHIS-1	TCCCAGAAAAAGAGGCAGAA
QHIS-2	TTCAGTGGTGTGATGGTCGT
QCM-1	TGGAGTGAATACCACGACGA
QCM-2	AAACGGGGGCGAAGAAGT
QNR-1	CGAATGGGCTGCTAACTTTC
QNR-2	TAAACATCATCGCCTGCTTC
QUREA-1	GACCTTTCTGGATGGGACAA
QUREA-2	ATTGGCGTTGATGGTAATGG
QRBCS-1	CGACCACCTTCTGGATTAGC
QRBCS-2	TGCATTTAACGCTGCTTACG
QCYTB-1	TCGTACATGGAACCCAGACA
QCYTB-2	TGGTTGACCTGCAATAGGAA
PNORM1-1	TTTCCCAGTCACGACGTTGTAAAACGACGGCCAGTGAAGCGTATTACAAATGAAACCAAG
PNORM1-2	CAAGGTGCTGATGCCGCTGGCGATTCAGGTTCAAATCCTGATCCAAACCTTTTTACTC
PNORM1-3	TACTGGCGCGTGGAGTAAAAAGGTTTGGATCAGGATTTGAACCTGAATCGCCAGC
PNORM1-4	ACATTTGTGTAGGGTCTTGTGGGTTTCGCAAAATGAGCTGGTGATATGGGATAGTGTT

PNORM1-5	GTAACAAGGGTGAACACTATCCCATATCACCAGCTCATTTTGCGAAACCCACAAGAC
PNORM1-6	TCAGCAACCGAATCCTGTCCGTCCCGAATCTTGCTATTGAAAATGTATTGTAAAGTGC
PNORM1-7	TATCAATTTTGCACTTTACAATACATTTTCAATAGCAAGATTCGGGACGGACAGGATT
PNORM1-8	TTCACACAGGAAACAGCTATGACCATGATTACGCCAGAAACGTACAGACGCTCCCGA
PNORM2-1	TTCCCAGTCACGACGTTGTAAAACGACGGCCAGTGTTAGTAACGACCACCTTCTGGAT
PNORM2-2	ACCCATTTTTTACATCTCTCATAATATATTCTACTGAGACTTACACAAGGTTGCTTCT
PNORM2-3	AACGAGAAGCAACCTTGTGTAAGTCTCAGTAGAATATATTATGAGAGATGTAAAAAATGG
PNORM2-4	ACACAGGAAACAGCTATGACCATGATTACGCCAAAGTTAAATGAAGTAAAGATACGCCTG
SB2	GACTTCGTGGAGGACGACTT
3'SHBLE	GTCCTGCTCCTCGGCCA
TP-PEPCK-F	CACCATGATTGCCACGCAAAGCCG
TP-PEPCK-R	TAAGATCTTTGGCCCATAGATT
TPCONJ-F1:	ATAACACTAGCTCGTCTTCACGTAACTATAACGGTCCTAATTGAGCTAGTCAGTCATCGCCTAGCTTCGGA
TPCONJ-R1:	AGCAGGGTTATGCAGCGGAAGATCTATATTACCCTGTTATTGGCGGCCGGCCGCTCTAGAACTAGTGGATC
TP-PEPCK-F	CACCATGATTGCCACGCAAAGCCG
TP-PEPCK-R	TAAGATCTTTGGCCCATAGATT
TPCONJ-F1	ATAACACTAGCTCGTCTTCACGTAACTATAACGGTCCTAATTGAGCTAGTCAGTCATCGCCTAGCTTCGGA
TPCONJ-R1	AGCAGGGTTATGCAGCGGAAGATCTATATTACCCTGTTATTGGCGGCCGGCCGCTCTAGAACTAGTGGATC
Primers to assemble vectors for cloning F1, F2, F3, F4, F5	
C25F1_H1F	CGTATCGTGAGCATCCTCTCTCGTTTCATCGCTTAATTAAGACTAATTGCAGCTTCTTCT
C25F1_H1R	TAGACGGCCGCCAGCCCAGCGGCGAGGGCAACCAGCTCGACCGAGTCTATTCGTCGATGG
C25F1_H2F	GAGCTGTAAGTACATCACCGACGAGCAAGGCAAGACGATCCGCGCCTGTTTTATTGAGAA
C25F1_H2R	TCATCTACCTGCCTGGACAGCATGGCCTGCAACGCTCGAGTCCGTTACCGTGGGGCCATG
C25F2_H1F	CGTATCGTGAGCATCCTCTCTCGTTTCATCGCTTAATTAAAATGCGGGTAGAGCCGTCGG
C25F2_H1R	TAGACGGCCGCCAGCCCAGCGGCGAGGGCAACCAGCTCGACCGCCCTGAACGACTTCTAG
C25F2_H2F	GAGCTGTAAGTACATCACCGACGAGCAAGGCAAGACGATCCGCGCCCCATGGCGTTTGCG
C25F2_H2R	TCATCTACCTGCCTGGACAGCATGGCCTGCAACGCTCGAGAGATCTTCTTCTGTTGCAGG
C25F3_H1F	CGTATCGTGAGCATCCTCTCTCGTTTCATCGCTTAATTAACTGAGATGAATGCTAAATAC
C25F3_H1R	TAGACGGCCGCCAGCCCAGCGGCGAGGGCAACCAGCTCGACCAGTGATCGTGGCGGATGG
C25F3_H2F	GAGCTGTAAGTACATCACCGACGAGCAAGGCAAGACGATCCGCGCCCTTTGTCATGTTCG
C25F3_H2R	TCATCTACCTGCCTGGACAGCATGGCCTGCAACGCTCGAGTGGGTTCCTTGTCGACGGCC
C25F4_H1F	CGTATCGTGAGCATCCTCTCTCGTTTCATCGCTTAATTAACATCGTTCACAACCGTTGGC
C25F4_H1R	TAGACGGCCGCCAGCCCAGCGGCGAGGGCAACCAGCTCGAATCTACTAAACCAACCGTGA
C25F4_H2F	GAGCTGTAAGTACATCACCGACGAGCAAGGCAAGACGATCCGCGCCCAGCGCAATGTGCG
C25F4_H2R	TCATCTACCTGCCTGGACAGCATGGCCTGCAACGCTCGAGTCCCTCAGGCCACCTTTTCT
C25F5_H1F	CGTATCGTGAGCATCCTCTCTCGTTTCATCGCTTAATTAACGGAAACGAGCCACAGCATT
C25F5_H1R	TAGACGGCCGCCAGCCCAGCGGCGAGGGCAACCAGCTCGACCTTGTGTGTACTGCCCACC
C25F5_H2F	GAGCTGTAAGTACATCACCGACGAGCAAGGCAAGACGATCGATAGCGGGGAAAAACACGA
C25F5_H2R	TCATCTACCTGCCTGGACAGCATGGCCTGCAACGCTCGAGTATTGCTGTCATAGCATCAC
Primers to screen for correctly assembled plasmids containing F1, F2. F3, F4, F5	
MPXpt25_F1	GTTCTTTCCGAAGCGTTGAG
MPXpt25_R1	TCTTCGTGAAAGGCTTCGTT
MPXpt25_F2	CGACAGGAGGAATAGCGAAG
MPXpt25_R2	CTGGAAGGTGGTTGTGGAGT
MPXpt25_F3	CTTTGTCGCCTGCAGTGTTA
MPXpt25_R3	TGGTCAGTCCTCGATTAGGG
MPXpt25_F4	TTGGGATGCTGAACAATCAA
MPXpt25_R4	AAATCACCGATGACCCGTTA
MPXpt25_F5	ACGAATGCGAAGAGCAAACT
MPXpt25_R5	ACACGATCGAAACGAGATCC
Primers to assemble vectors for cloning p0521-Se	
SeHook1_F	CGTAACTATAACGGTCCTAACCAAGCCTCGTTGGCTAGGTAGCGCCAGCGAGGCAGATGC
SeHook1_R	GCCTGCAACGTTCGCTACCctcgagCGGTGATTTTGCTGGCGCTGACTGGTTTTTCGATT
SeHook2_F	CGAGTTACGCTAGGGATAActcgagACCAGTTGATCGACGAGCTTTTCGCCTAGACCTTC
SeHook2_R	CTATATTACCCTGCCGGCCGATGCTAAACCTGTCGTGCTGCCGGAGAAGTGTC
Primers to screen for correct assembly of p0521Se with 48,602 kb S. elongatus fragment	
pS0521MpIIF1	AGTTGCAGTCACTCCGCTTT
pS0521MpIIR1	GGATCCGTTTGAAGATCACG
pS0521MpIIF2	ACCAGTTGATCGACGAGCTT
pS0521MpIIR2	GGTGCGAATAAGGGACAGTG
pS0521MpIIF3	CTTGCAGCAAATCCAAGACA
pS0521MpIIR3	TGGGTCAGCAACTCAGACAG

Primers to insert URA3 flanked by I-CeuI and I-SceI sites	
0521URA_F	GCTTTGGTTTCACAGTCAGGAATAACACTAGCTCGTCTTCACGTAACTATAACGGTCCTAAGGTAGCGAACGTTGCAGGCCATGCTGTCC
0521URA_R	GCTATAATGACCCCGAAGCAGGGTTATGCAGCGGAAGATGCTATATTACCCTGTTATCCCTAGCGTAACTCGATGAAACGAGAGAGGATG

Supplementary Table 2: Optimization of conjugation to *P. tricornutum*

Exp. #	*P. tricornutum* culture characteristics: Cell culture (L = liquid, P = plates), F = chlorophyll A autofluorescence (for liquid cultures only), Cell morphotype (fusiform, ovoid, triradiate), Final concentration of cells, Amount used	Cargo Plasmid used	*E. coli* culture OD_{600}, amount used in conjugation reaction, used directly or concentrated in SOC media	Initial plates during conjug-ation	Time at 30°C for conjug-ation	Time at 18°C after 30 °C and before selection	How much scraped cells was plated on selection plates	Number of ex-conjugant colonies (after 14 days or more)
1	L, F ~200, fusiforms, Final $1.0x10^8$, 200µl	p0521s	0.31, 400µl (directly)	L1, 5%LB	120 min	1 day	400 µl	0
2	L, F ~200, fusiforms, Final $1.0x10^8$, 200µl	p0521s	0.31, 400µl (directly)	L1, 5%LB	120 min	1 day	400 µl	0
3	L, F ~200, fusiforms, Final $1.0x10^8$, 200µl	p0521s	0.31, 400µl (directly)	L1, 5%LB	120 min	1 day	800 µl	0
4	L, F ~200, fusiforms, Final $1.0x10^8$, 200µl	p0521s	0.31, 400µl (directly)	L1, 5%LB	120 min	1 day	800 µl	2
5	L, F ~200, fusiforms, Final $1.0x10^8$, 200µl	p0521s	0.31, 400µl (directly)	L1, 5%LB	120 min	2 days	400 µl	5
6	L, F ~200, fusiforms, Final $1.0x10^8$, 200µl	p0521s	0.31, 400µl (directly)	L1, 5%LB	120 min	2 days	400 µl	0
7	L, F ~200, fusiforms, Final $1.0x10^8$, 200µl	p0521s	0.31, 400µl (directly)	L1, 5%LB	120 min	2 days	800 µl	4
8	L, F ~200, fusiforms, Final $1.0x10^8$, 200µl	p0521s	0.31, 400µl (directly)	L1, 5%LB	120 min	2 days	800 µl	8
9	P, 70% fusiform, 30% oval, Final $1.0x10^8$, 200µl	p0521s	0.31, 400µl (directly)	L1, 5%LB	120 min	1 day	400 µl	4
10	P, 70% fusiform, 30% oval, Final $1.0x10^8$, 200µl	p0521s	0.31, 400µl (directly)	L1, 5%LB	120 min	1 day	400 µl	1
11	P, 70% fusiform, 30% oval, Final $1.0x10^8$, 200µl	p0521s	0.31, 400µl (directly)	L1, 5%LB	120 min	1 day	800 µl	4
12	P, 70% fusiform, 30% oval, Final $1.0x10^8$, 200µl	p0521s	0.31, 400µl (directly)	L1, 5%LB	120 min	1 day	800 µl	0
13	P, 70% fusiform, 30% oval, Final 1.0x108, 200µl	p0521s	0.31, 400µl (directly)	L1, 5%LB	120 min	2 days	400 µl	5
14	P, 70% fusiform, 30% oval, Final $1.0x10^8$, 200µl	p0521s	0.31, 400µl (directly)	L1, 5%LB	120 min	2 days	400 µl	3
15	P, 70% fusiform, 30% oval, Final $1.0x10^8$, 200µl	p0521s	0.31, 400µl (directly)	L1, 5%LB	120 min	2 days	800 µl	4
16	P, 70% fusiform, 30% oval, Final $1.0x10^8$, 200µl	p0521s	0.31, 400µl (directly)	L1, 5%LB	120 min	2 days	800 µl	6
17	L, F ~200, fusiform, Final $1.0x10^8$, 300µl	p0521s	0.41, 300µl (directly)	L1 only	120 min	1 day	800 µl	0
18	L, F ~200, fusiform, Final $1.0x10^8$, 300µl	p0521s	0.41, 300µl (directly)	L1 only	120 min	1 day	800 µl	1
19	L, F ~200, fusiform, Final $1.0x10^8$, 300µl	p0521s	0.41, 300µl (directly)	L1, 5%LB	120 min	1 day	800 µl	1
20	L, F ~200, fusiform, Final $1.0x10^8$, 300µl	p0521s	0.41, 300µl (directly)	L1, 5%LB	120 min	1 day	800 µl	0
21	L, F ~200, fusiform, Final $1.0x10^8$, 300µl	p0521s	0.41, 300µl (directly)	L1, 10%LB	120 min	1 day	800 µl	1
22	L, F ~200, fusiform, Final $1.0x10^8$, 300µl	p0521s	0.41, 300µl (directly)	L1, 10%LB	120 min	1 day	800 µl	1
23	L, F ~200, fusiform, Final $1.0x10^8$, 300µl	p0521s	0.41, 300µl (directly)	L1, 20%LB	120 min	1 day	800 µl	2
24	L, F ~200, fusiform, Final $1.0x10^8$, 300µl	p0521s	0.41, 300µl (directly)	L1, 20%LB	120 min	1 day	800 µl	1
25	L, F ~200, fusiform, Final $1.0x10^8$, 200µl	p0521s	0.38, 400µl (directly)	L1, 5%LB	30 min	1 day	800 µl	2
26	L, F ~200, fusiform, Final $1.0x10^8$, 200µl	p0521s	0.38, 400µl (directly)	L1, 5%LB	30 min	1 day	800 µl	1
27	L, F ~200, fusiform, Final $1.0x10^8$, 200µl	p0521s	0.38, 400µl (directly)	L1, 5%LB	60 min	1 day	800 µl	4
28	L, F ~200, fusiform, Final $1.0x10^8$, 200µl	p0521s	0.38, 400µl (directly)	L1, 5%LB	60 min	1 day	800 µl	3
29	L, F ~200, fusiform, Final $1.0x10^8$, 200µl	p0521s	0.38, 400µl (directly)	L1, 5%LB	90 min	1 day	800 µl	3
30	L, F ~200, fusiform, Final $1.0x10^8$, 200µl	p0521s	0.38, 400µl (directly)	L1, 5%LB	90 min	1 day	800 µl	10
31	L, F ~200, fusiform, Final $1.0x10^8$, 200µl	p0521s	0.38, 400µl (directly)	L1, 5%LB	120 min	1 day	800 µl	2
32	L, F ~200, fusiform, Final $1.0x10^8$, 200µl	p0521s	0.38, 400µl (directly)	L1, 5%LB	120 min	1 day	800 µl	6
33	L, F ~25, fusiform, Final $0.9x10^8$, 100µl	p0521s	0.40, 50µl (directly)	L1, 5%LB	120 min	1 day	800 µl	0
34	L, F ~25, fusiform, Final $0.9x10^8$, 100µl	p0521s	0.40, 100µl (directly)	L1, 5%LB	120 min	1 day	800 µl	2
35	L, F ~25, fusiform, Final $0.9x10^8$, 100µl	p0521s	0.40, 150µl (directly)	L1, 5%LB	120 min	1 day	800 µl	1

36	L, F ~25, fusiform, Final 0.9x10^{8}, 100µl	p0521s	0.40, 200µl (directly)	L1, 5%LB	120 min	1 day	800 µl	3
37	L, F ~25, fusiform, Final 0.9x10^{8}, 200µl	p0521s	0.40, 50µl (directly)	L1, 5%LB	120 min	1 day	800 µl	1
38	L, F ~25, fusiform, Final 0.9x10^{8}, 200µl	p0521s	0.40, 100µl (directly)	L1, 5%LB	120 min	1 day	800 µl	2
39	L, F ~25, fusiform, Final 0.9x10^{8}, 200µl	p0521s	0.40, 150µl (directly)	L1, 5%LB	120 min	1 day	800 µl	3
40	L, F ~25, fusiform, Final 0.9x10^{8}, 200µl	p0521s	0.40, 200µl (directly)	L1, 5%LB	120 min	1 day	800 µl	5
41	L, F ~25, fusiform, Final 1.8x10^{8}, 200µl	p0521s	1.05, 200µl -25mL LB into 1 ml SOC	L1, 5%LB	90 min	1 day	800 µl	73
42	L, F ~25, fusiform, Final 1.8x10^{8}, 200µl	p0521s	1.05, 200µl -25mL LB into 1 ml SOC	L1, 5%LB	90 min	1 day	800 µl	109
43	L, F ~25, fusiform, Final 1.8x10^{8}, 200µl	p0521s	0.88, 200µl -25mL LB into 1 ml SOC	L1, 5%LB	90 min	1 day	800 µl	74
44	L, F ~25, fusiform, Final 1.8x10^{8}, 200µl	p0521s	0.88, 200µl -25mL LB into 1 ml SOC	L1, 5%LB	90 min	1 day	800 µl	86
45	L, F ~25, fusiform, Final 1.8x10^{8}, 200µl	p0521s	0.68, 200µl -25mL LB into 1 ml SOC	L1, 5%LB	90 min	1 day	800 µl	42
46	L, F ~25, fusiform, Final 1.8x10^{8}, 200µl	p0521s	0.68, 200µl -25mL LB into 1 ml SOC	L1, 5%LB	90 min	1 day	800 µl	75
47	L, F ~25, fusiform, Final 1.8x10^{8}, 200µl	p0521s	0.47, 200µl -25mL LB into 1 ml SOC	L1, 5%LB	90 min	1 day	800 µl	57
48	L, F ~25, fusiform, Final 1.8x10^{8}, 200µl	p0521s	0.47, 200µl -25mL LB into 1 ml SOC	L1, 5%LB	90 min	1 day	800 µl	41
49	L, F ~25, fusiform, Final 1.8x10^{8}, 200µl	p0521s	0.20, 200µl -25mL LB into 1 ml SOC	L1, 5%LB	90 min	1 day	800 µl	47
50	L, F ~25, fusiform, Final 1.8x10^{8}, 200µl	p0521s	0.20, 200µl -25mL LB into 1 ml SOC	L1, 5%LB	90 min	1 day	800 µl	42
51	P, fusiform, 20% oval or in transition to oval, Final 2.0 x10^{8}, 200µl	p0521s	0.58, 200µl -90 mL LB into 1.8 mL SOC	L1	90 min	1 day	400 µl	11
52	P, fusiform, 20% oval or in transition to oval, Final 2.0 x10^{8}, 200µl	p0521s	0.58, 200µl -90 mL LB into 1.8 mL SOC	L1	90 min	1 day	400 µl	12
53	P, fusiform, 20% oval or in transition to oval, Final 2.0 x10^{8}, 200µl	p0521s	0.58, 200µl -90 mL LB into 3.6 mL SOC	L1	90 min	1 day	400 µl	28
54	P, fusiform, 20% oval or in transition to oval, Final 2.0 x10^{8}, 200µl	p0521s	0.58, 200µl -90 mL LB into 3.6 mL SOC	L1	90 min	1 day	400 µl	6
55	P, fusiform, 20% oval or in transition to oval, Final 2.0 x10^{8}, 200µl	p0521s	0.58, 200µl -90 mL LB into 1.8 mL SOC	L1, 5%LB	90 min	1 day	400 µl	52
56	P, fusiform, 20% oval or in transition to oval, Final 2.0 x10^{8}, 200µl	p0521s	0.58, 200µl -90 mL LB into 1.8 mL SOC	L1, 5%LB	90 min	1 day	400 µl	42
57	P, fusiform, 20% oval or in transition to oval, Final 2.0 x10^{8}, 200µl	p0521s	0.58, 200µl -90 mL LB into 3.6 mL SOC	L1, 5%LB	90 min	1 day	400 µl	106
58	P, fusiform, 20% oval or in transition to oval, Final 2.0 x10^{8}, 200µl	p0521s	0.58, 200µl -90 mL LB into 3.6 mL SOC	L1, 5%LB	90 min	1 day	400 µl	103
59	P, fusiform, 20% oval or in transition to oval, Final 2.0 x10^{8}, 200µl	p0521s	0.58, 200µl -90 mL LB into 1.8 mL SOC	L1, 10%LB	90 min	1 day	400 µl	33
60	P, fusiform, 20% oval or in transition to oval, Final 2.0 x10^{8}, 200µl	p0521s	0.58, 200µl -90 mL LB into 1.8 mL SOC	L1, 10%LB	90 min	1 day	400 µl	30
61	P, fusiform, 20% oval or in transition to oval, Final 2.0 x10^{8}, 200µl	p0521s	0.58, 200µl -90 mL LB into 3.6 mL SOC	L1, 10%LB	90 min	1 day	400 µl	43
62	P, fusiform, 20% oval or in transition to oval, Final 2.0 x10^{8}, 200µl	p0521s	0.58, 200µl -90 mL LB into 3.6 mL SOC	L1, 10%LB	90 min	1 day	400 µl	49
63	P, fusiform, 20% oval or in transition to oval, Final 2.0 x10^{8}, 200µl	p0521s	0.58, 200µl -90 mL LB into 1.8 mL SOC	L1, 20%LB	90 min	1 day	400 µl	51
64	P, fusiform, 20% oval or in transition to oval, Final 2.0 x10^{8}, 200µl	p0521s	0.58, 200µl -90 mL LB into 1.8 mL SOC	L1, 20%LB	90 min	1 day	400 µl	90
65	P, fusiform, 20% oval or in transition to oval, Final 2.0 x10^{8}, 200µl	p0521s	0.58, 200µl -90 mL LB into 3.6 mL SOC	L1, 20%LB	90 min	1 day	400 µl	52
66	P, fusiform, 20% oval or in transition to oval, Final 2.0 x10^{8}, 200µl	p0521s	0.58, 200µl -90 mL LB into 3.6 mL SOC	L1, 20%LB	90 min	1 day	400 µl	46
67	P, fusiform, 20% oval or in transition to oval, Final 6.0 x10^{8}, 200µl	p0521s	0.58, 50µl -90 mL LB into 3.6 mL SOC	L1, 5%LB	90 min	1 day	400 µl	4
68	P, fusiform, 20% oval or in transition to oval, Final 5.0 x10^{8}, 200µl	p0521s	0.58, 50µl -90 mL LB into 3.6 mL SOC	L1, 5%LB	90 min	1 day	400 µl	0
69	P, fusiform, 20% oval or in transition to oval, Final 4.0 x10^{8}, 200µl	p0521s	0.58, 50µl -90 mL LB into 3.6 mL SOC	L1, 5%LB	90 min	1 day	400 µl	0
70	P, fusiform, 20% oval or in transition to oval, Final 3.0 x10^{8}, 200µl	p0521s	0.58, 50µl -90 mL LB into 3.6 mL SOC	L1, 5%LB	90 min	1 day	400 µl	0
71	P, fusiform, 20% oval or in transition to oval, Final 2.0 x10^{8}, 200µl	p0521s	0.58, 50µl -90 mL LB into 3.6 mL SOC	L1, 5%LB	90 min	1 day	400 µl	1
72	P, fusiform, 20% oval or in transition to oval, Final 2.0 x10^{8}, 100µl	p0521s	0.52, 100µl, 200 mL LB into 6 mL SOC	L1, 5%LB	90 min	1 day	400 µl	112

73	P, fusiform, 20% oval or in transition to oval, Final 2.0 x10^8, 100µl	p0521s	0.52, 100µl, 200 mL LB into 6 mL SOC	L1, 10%LB	90 min	1 day	400 µl	38
74	P, fusiform, 20% oval or in transition to oval, Final 2.0 x10^8, 100µl	p0521s	0.52, 100µl, 200 mL LB into 6 mL SOC	L1, 20%LB	90 min	1 day	400 µl	53
75	P, fusiform, 20% oval or in transition to oval, Final 2.0 x10^8, 100µl	p0521s	0.52, 200µl, 200 mL LB into 6 mL SOC	L1, 5%LB	90 min	1 day	400 µl	145
76	P, fusiform, 20% oval or in transition to oval, Final 2.0 x10^8, 100µl	p0521s	0.52, 200µl, 200 mL LB into 6 mL SOC	L1, 10%LB	90 min	1 day	400 µl	70
77	P, fusiform, 20% oval or in transition to oval, Final 2.0 x10^8, 100µl	p0521s	0.52, 200µl, 200 mL LB into 6 mL SOC	L1, 20%LB	90 min	1 day	400 µl	46
78	P, fusiform, 20% oval or in transition to oval, Final 2.0 x10^8, 200µl	p0521s	0.52, 100µl, 200 mL LB into 6 mL SOC	L1, 5%LB	90 min	1 day	400 µl	203
79	P, fusiform, 20% oval or in transition to oval, Final 2.0 x10^8, 200µl	p0521s	0.52, 100µl, 200 mL LB into 6 mL SOC	L1, 10%LB	90 min	1 day	400 µl	57
80	P, fusiform, 20% oval or in transition to oval, Final 2.0 x10^8, 200µl	p0521s	0.52, 100µl, 200 mL LB into 6 mL SOC	L1, 20%LB	90 min	1 day	400 µl	106
81	P, fusiform, 20% oval or in transition to oval, Final 2.0 x10^8, 200µl	p0521s	0.52, 200µl, 200 mL LB into 6 mL SOC	L1, 5%LB	90 min	1 day	400 µl	332
82	P, fusiform, 20% oval or in transition to oval, Final 2.0 x10^8, 200µl	p0521s	0.52, 200µl, 200 mL LB into 6 mL SOC	L1, 10%LB	90 min	1 day	400 µl	260
83	P, fusiform, 20% oval or in transition to oval, Final 2.0 x10^8, 200µl	p0521s	0.52, 200µl, 200 mL LB into 6 mL SOC	L1, 20%LB	90 min	1 day	400 µl	121
84	P, fusiform, 20% oval or in transition to oval, Final 4.0 x10^8, 100µl	p0521s	0.52, 100µl, 200 mL LB into 6 mL SOC	L1, 5%LB	90 min	1 day	400 µl	169
85	P, fusiform, 20% oval or in transition to oval, Final 4.0 x10^8, 100µl	p0521s	0.52, 100µl, 200 mL LB into 6 mL SOC	L1, 10%LB	90 min	1 day	400 µl	210
86	P, fusiform, 20% oval or in transition to oval, Final 4.0 x10^8, 100µl	p0521s	0.52, 100µl, 200 mL LB into 6 mL SOC	L1, 20%LB	90 min	1 day	400 µl	111
87	P, fusiform, 20% oval or in transition to oval, Final 4.0 x10^8, 100µl	p0521s	0.52, 200µl, 200 mL LB into 6 mL SOC	L1, 5%LB	90 min	1 day	400 µl	311
88	P, fusiform, 20% oval or in transition to oval, Final 4.0 x10^8, 100µl	p0521s	0.52, 200µl, 200 mL LB into 6 mL SOC	L1, 10%LB	90 min	1 day	400 µl	215
89	P, fusiform, 20% oval or in transition to oval, Final 4.0 x10^8, 100µl	p0521s	0.52, 200µl, 200 mL LB into 6 mL SOC	L1, 20%LB	90 min	1 day	400 µl	117
90	P, fusiform, 20% oval or in transition to oval, Final 4.0 x10^8, 200µl	p0521s	0.52, 100µl, 200 mL LB into 6 mL SOC	L1, 5%LB	90 min	1 day	400 µl	247
91	P, fusiform, 20% oval or in transition to oval, Final 4.0 x10^8, 200µl	p0521s	0.52, 100µl, 200 mL LB into 6 mL SOC	L1, 10%LB	90 min	1 day	400 µl	280
92	P, fusiform, 20% oval or in transition to oval, Final 4.0 x10^8, 200µl	p0521s	0.52, 100µl, 200 mL LB into 6 mL SOC	L1, 20%LB	90 min	1 day	400 µl	211
93	P, fusiform, 20% oval or in transition to oval, Final 4.0 x10^8, 200µl	p0521s	0.52, 200µl, 200 mL LB into 6 mL SOC	L1, 5%LB	90 min	1 day	400 µl	460
94	P, fusiform, 20% oval or in transition to oval, Final 4.0 x10^8, 200µl	p0521s	0.52, 200µl, 200 mL LB into 6 mL SOC	L1, 10%LB	90 min	1 day	400 µl	450
95	P, fusiform, 20% oval or in transition to oval, Final 4.0 x10^8, 200µl	p0521s	0.52, 200µl, 200 mL LB into 6 mL SOC	L1, 20%LB	90 min	1 day	400 µl	263
96	P, fusiform, 20% oval or in transition to oval, Final 6.0 x10^8, 100µl	p0521s	0.52, 100µl, 200 mL LB into 6 mL SOC	L1, 5%LB	90 min	1 day	400 µl	226
97	P, fusiform, 20% oval or in transition to oval, Final 6.0 x10^8, 100µl	p0521s	0.52, 100µl, 200 mL LB into 6 mL SOC	L1, 10%LB	90 min	1 day	400 µl	226
98	P, fusiform, 20% oval or in transition to oval, Final 6.0 x10^8, 100µl	p0521s	0.52, 100µl, 200 mL LB into 6 mL SOC	L1, 20%LB	90 min	1 day	400 µl	173
99	P, fusiform, 20% oval or in transition to oval, Final 6.0 x10^8, 100µl	p0521s	0.52, 200µl, 200 mL LB into 6 mL SOC	L1, 5%LB	90 min	1 day	400 µl	532
100	P, fusiform, 20% oval or in transition to oval, Final 6.0 x10^8, 100µl	p0521s	0.52, 200µl, 200 mL LB into 6 mL SOC	L1, 10%LB	90 min	1 day	400 µl	385
101	P, fusiform, 20% oval or in transition to oval, Final 6.0 x10^8, 100µl	p0521s	0.52, 200µl, 200 mL LB into 6 mL SOC	L1, 20%LB	90 min	1 day	400 µl	234
102	P, fusiform, 20% oval or in transition to oval, Final 6.0 x10^8, 200µl	p0521s	0.52, 100µl, 200 mL LB into 6 mL SOC	L1, 5%LB	90 min	1 day	400 µl	232
103	P, fusiform, 20% oval or in transition to oval, Final 6.0 x10^8, 200µl	p0521s	0.52, 100µl, 200 mL LB into 6 mL SOC	L1, 10%LB	90 min	1 day	400 µl	238
104	P, fusiform, 20% oval or in transition to oval, Final 6.0 x10^8, 200µl	p0521s	0.52, 100µl, 200 mL LB into 6 mL SOC	L1, 20%LB	90 min	1 day	400 µl	216
105	P, fusiform, 20% oval or in transition to oval, Final 6.0 x10^8, 200µl	p0521s	0.52, 200µl, 200 mL LB into 6 mL SOC	L1, 5%LB	90 min	1 day	400 µl	1100
106	P, fusiform, 20% oval or in transition to oval, Final 6.0 x10^8, 200µl	p0521s	0.52, 200µl, 200 mL LB into 6 mL SOC	L1, 10%LB	90 min	1 day	400 µl	618
107	P, fusiform, 20% oval or in transition to oval, Final 6.0 x10^8, 200µl	p0521s	0.52, 200µl, 200 mL LB into 6 mL SOC	L1, 20%LB	90 min	1 day	400 µl	458

108	P, Final $5.8x10^{8}$, 200µl	p0521s	0.66, 200µl, 20 mL LB into 0.5 mL SOC	L1, 5%LB	90 min	2 days	3x300 µl	5660
109	P, Final $5.8x10^{8}$, 200µl	p0521s ΔR1	0.60, 200µl, 20 mL LB into 0.5 mL SOC	L1, 5%LB	90 min	2 days	3x300 µl	2894
110	P, Final $5.8x10^{8}$, 200µl	p0521s ΔR12	0.48, 200µl, 20 mL LB into 0.5 mL SOC	L1, 5%LB	90 min	2 days	3x300 µl	895
111	P, Final $5.8x10^{8}$, 200µl	p0521s ΔR2	0.54, 200µl, 20 mL LB into 0.5 mL SOC	L1, 5%LB	90 min	2 days	3x300 µl	2393
112	P, Final $5.8x10^{8}$, 200µl	p0521s ΔoriT	0.60, 200µl, 20 mL LB into 0.5 mL SOC	L1, 5%LB	90 min	2 days	3x300 µl	0

Supplementary Table 3: Summary of data

Plasmid used in conjugation	Total number of *P. tricornutum* colonies	Number of *P. tricornutum* colonies from which plasmid were rescued in *E. coli*	Number of *P. tricornutum* colonies from which correct plasmids were rescued in *E.coli*
p0521s/pRL443	5,660	16/20	7/20
p0521s ΔR1R2 /pRL443	895	18/20	8/20
p0521s/pTA-MOB	10,628	18/20	14/20
p0521s ΔR1R2/pTA-MOB	4,915	17/20	11/20
p0521Se/pTA-MOB	1,900	17/20	7/20
pPtPuc1/pTA-MOB	2,750	20/20	8/20
pPtPuc2/pTA-MOB	64 large, 373 small	0/20	0/20
pPtPuc3/pTA-MOB	2,130	19/20	10/20
pPtPuc4/pTA-MOB	31 large, 31 small	0/20	0/20
pTpPuc3/pTA-MOB	500	16/18	16/18
pTpPuc4/pTA-MOB	85	0/18	0/18

Supplementary Table 4: Maintenance of p0521 without selection

Experiment	Number of *P. tricornutum* colonies surviving on plates (out of 100):	
	Without antibiotic selection	With antibiotic selection
1. *P. tricornutum* exconjugant line #3 passed for 28 days with antibiotic selection	100	93
2. *P. tricornutum* exconjugant line #3 passed for 28 days without antibiotic selection	100	27
3. *P. tricornutum* exconjugant line #9 passed for 28 days with antibiotic selection	100	90
4. *P. tricornutum* exconjugant line #9 passed for 28 days without antibiotic selection	100	42
